# Supplementary material for: Strategic land reallocation enhances carbon sequestration and biodiversity protection without compromising agricultural productivity in Great Britain
Source: Commun Earth Environ. 2025 Sep 29;6(1):770. doi: 10.1038/s43247-025-02728-w (PMC12479351; doi:10.1038/s43247-025-02728-w)
Supplement: Supplementary file 2 — Supplementary Information [file 43247_2025_2728_MOESM2_ESM.pdf]

Supplementary Information

For

Strategic land reallocation enhances carbon sequestration and biodiversity protection without compromising agricultural productivity in Great Britain

Sarah S. Gall[1, *], Tom Harwood[1], Michael Obersteiner[1], and Jim W. Hall[1]

Environmental Change Institute, University of Oxford, 3 S Parks Rd, Oxford OX1 3QY, United Kingdom
[*]Corresponding author: sarah.gall@spc.ox.ac.uk

Author Information

Affiliations

Environmental Change Institute, University of Oxford, 3 S Parks Rd, Oxford OX1 3QY, UK

Sarah S. Gall, Tom Harwood, Michael Obersteiner, and Jim W. Hall

Corresponding author

Correspondence to: Sarah S. Gall (sarah.gall@spc.ox.ac.uk) ORCID: 0000-0003-4676-4519

Supplementary Figures

Supplementary Figure 1 & Supplementary Figure 2 Testing of different step sizes – Pareto frontiers

These are the equivalents to Figure 1 with weightings step sizes 10 and 2 as a comparison (to step size 5 used in the analysis). While the optimisation in this paper generated 231 scenarios based on a step size of 5, step size 10 would only produce 66 scenarios, which are not sufficient to generate a smooth Pareto curve and step size 2 would produce 1326 scenarios while requiring significantly higher computational resources without providing improved outputs.

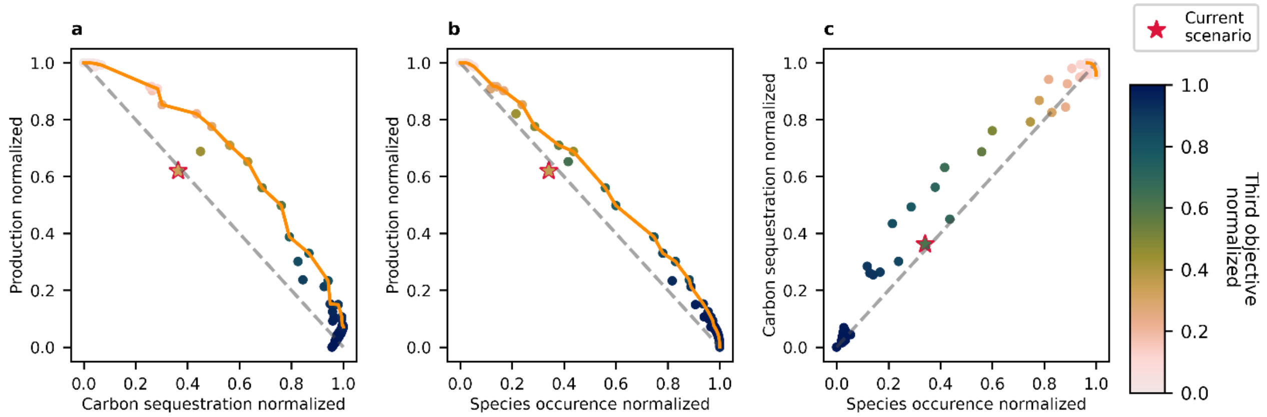

Supplementary Figure 1 Pareto frontiers resulting from a weighting step size of 10, including a total of 66 scenarios

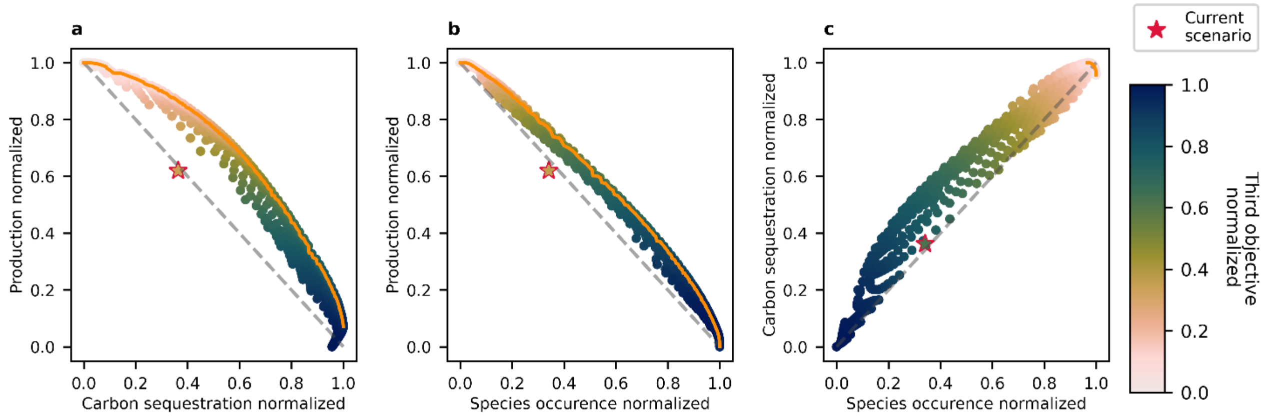

Supplementary Figure 2 Pareto frontier resulting from a weighting step size of 2, including a total of 1326 scenarios

Supplementary Figures 3 & 4 Testing of different step sizes – Hotspots of change

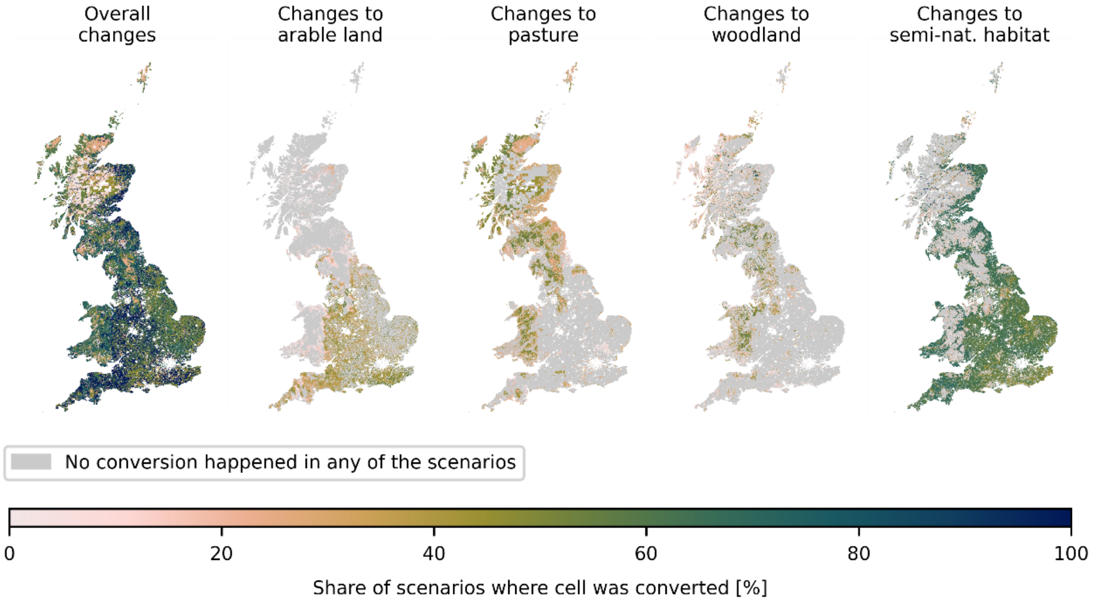

Supplementary Figure 3 Hotspots of change map created with a step size of 10, including a total of 66 scenarios

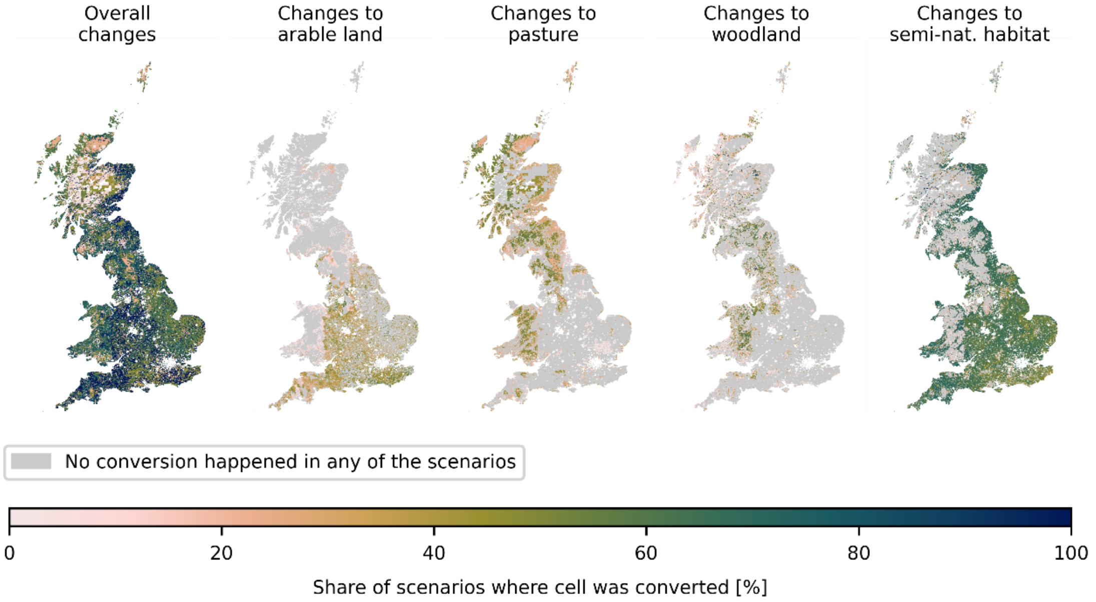

Supplementary Figure 4 Hotspots of change map created with a step size of 2, including a total of 1326 scenarios

Supplementary Figure 5 Example scenario maps

The following maps show how the spatial patterns differ for different priority weightings, showing the resulting landscapes for weightings 70-15-15 (carbon focused), 15-70-15 (production focused), 15-15-70 (biodiversity focused) and 30-35-35 (balanced) for the objectives carbon sequestration, production and biodiversity.

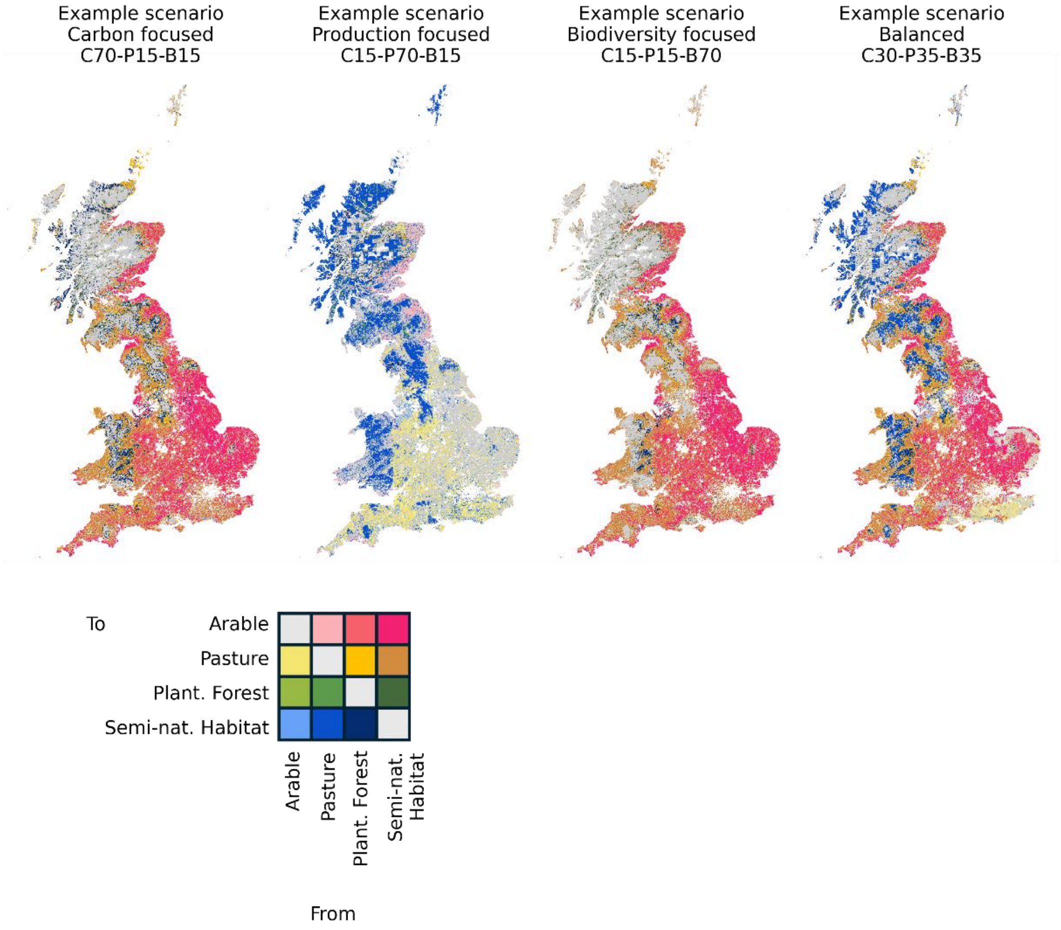

Supplementary Figure 5 Example scenario maps for weightings 70-15-15 (carbon focused), 15-70-15 (production focused), 15-15-70 (biodiversity focused) and 30-35-35 (balanced) for the objectives carbon sequestration, production and biodiversity

Supplementary Figure 6 Example scenario maps for increasing conversion budgets

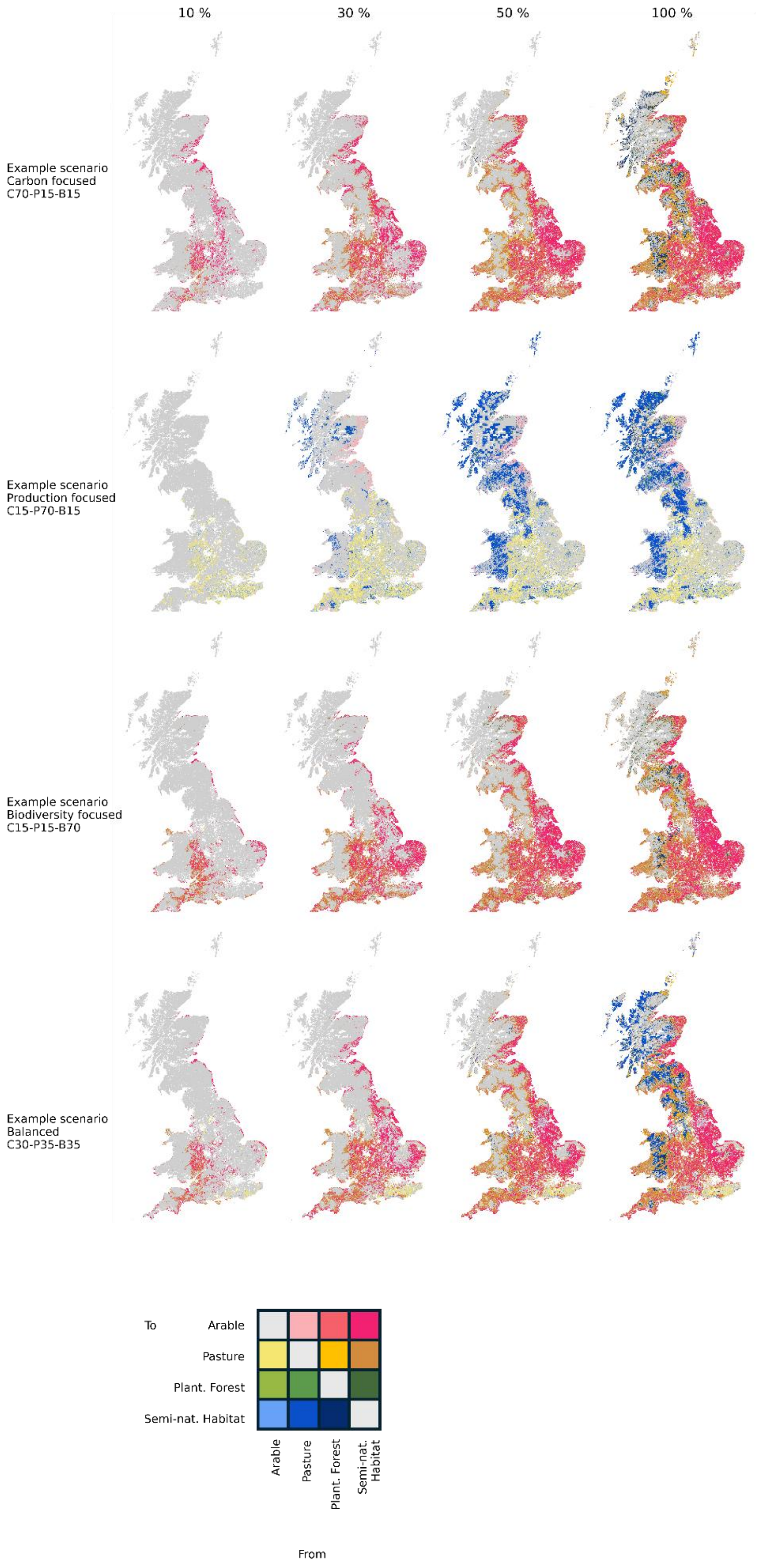

Supplementary Figure 6 Example scenario maps for weightings 70-15-15 (carbon focused), 15-70-15 (production focused), 15-15-70 (biodiversity focused) and 30-35-35 (balanced) and conversion budgets of 10%, 30%, 50% and 100% for the objectives carbon sequestration, production and biodiversity

Supplementary Figure 7 Land-use distribution resulting from different resampling methods

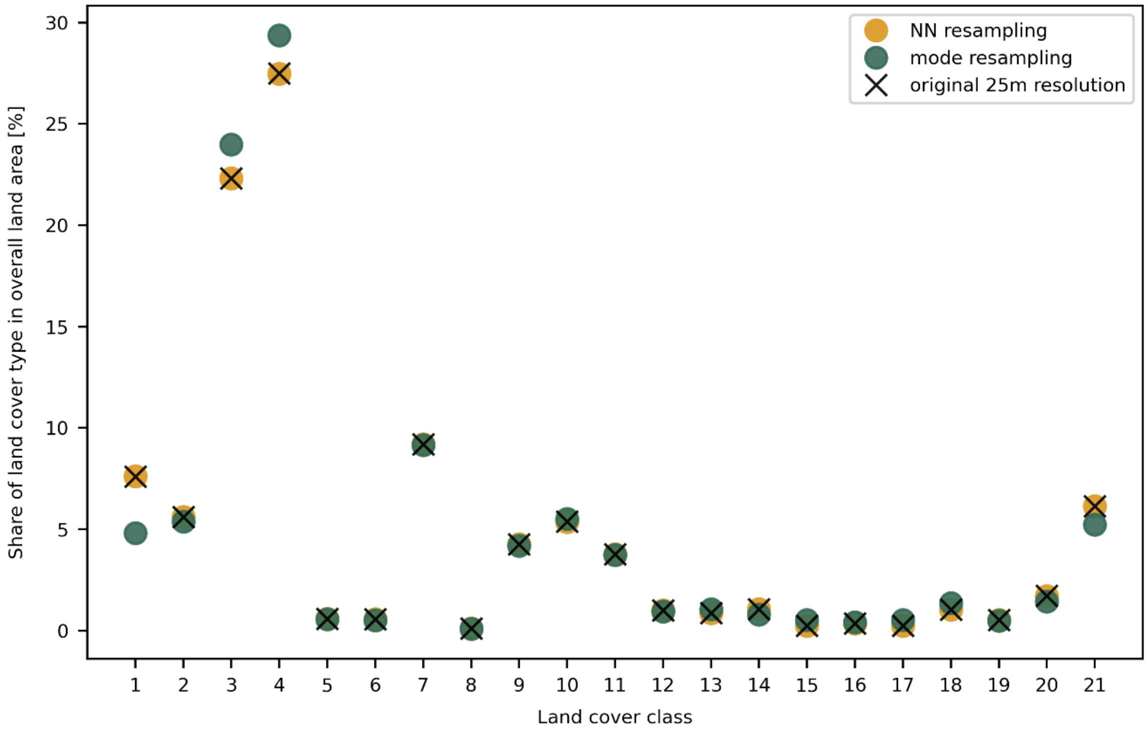

Supplementary Figure 7 Land cover distribution resulting from the resampling methods mode and nearest neighbour compared to the original distribution with a resolution (1: Deciduous woodland, 2: Coniferous woodland, 3: Arable, 4: Improved grassland, 5: Neutral grassland, 6: Calcareous grassland, 7: Acid grassland, 8: Fen, 9: Heather, 10: Heather grassland, 11: Bog, 12: Inland rock, 13: Saltwater, 14: Freshwater, 15: Supralittoral rock, 16: Supralittoral sediment, 17: Littoral rock, 18: Littoral sediment, 19: Saltmarsh, 20: Urban, 21: Suburban)

Supplementary Tables

Supplementary Table 1 Overview of existing land-use models

Model type & description	Pro's	Con's	Policy relevance & questions that can be answered	Comparison with our model
**Scenario calculators**<br>(e.g. FABLE calculator, CCC land-use scenarios)<br>based on exogenously specified land-use scenarios, which evaluate the consequences of those scenarios	• Give insights for land-use target setting<br>• Outline a few scenarios and compare them with a baseline	• Usually not spatially explicit<br>• Not suitable for exploring trade-offs	• What targets are achievable?<br>• How much do we have to reduce/increase certain land-use types and activities to meet a target?<br>• Gives insights for target setting and for calculating the potential effect of new strategies	• Evaluate only a small number of scenarios<br>• Usually not spatially explicit<br>• Not suitable for evaluating spatial trade-offs
**Agent-Based-models (ABMs)**<br>consider different groups of agents and their interactions with each other and with external drivers	• Insights into how agents react to changes in drivers/new developments	• Unsuitable for target setting<br>• Does not allow for identifying the land conversions that may be desirable in the first place	• What might be the effect of a new policy considering how different stakeholders react?<br>• Insights into how agents react to new policy interventions<br>• Testing of proposed policies for their potential impact	• ABMs are concerned with a different aspect of land-use policy research. They are designed to evaluate the behavioural dynamics of the actors, but they are not used for identifying targets or desirable outcomes in the first place
**Statistical/Empirical Models**<br>project future land-use patterns based on trends from historical data	• Predictions are based on evidence from the past<br>• Often spatially explicit	• Usually limited to projecting past patterns into the future and therefore not suitable for exploring policy shifts	• What do likely future land-use patterns look like based on past trends?<br>• Long-term projections and scenario analysis	• This type of model is concerned about what is likely to happen based on current trends, but is unsuitable for identifying desirable developments or, more specifically, preferable land conversions
**Global integrated land-use models**<br>(e.g. GLOBIOM, MagPIE)<br>Global land-use allocation models that combine economic	• Combine different sectors and economic and environmental aspects<br>• Often spatially explicit on a coarse level	• Often coarse spatial resolution or consideration of aggregated economic regions	• How should land be allocated globally to meet competing demands efficiently?<br>• What are the consequences of certain environmental targets on	• Less suitable for national policy making and simulating landscape features<br>• Not suitable for exploring spatial trade-offs on a fine resolution

and environmental aspects and are often spatially explicit			other aspects, such as food prices or deforestation?<br>• Insights into how changes to the land-use system affect other aspects	
**National Optimisation Models** (e.g. NEVO)<br>Optimise land-allocation by combining different objectives into one common metric such as the economic value	• Allocates land optimally based on the economic value of a range of ecosystem services<br>• Includes a large range of ecosystem services<br>• Spatially explicit	• By assigning a monetary value to all considered benefits (including water quality, recreational purposes, biodiversity, etc.), it implicitly assigns weightings that drive the optimisation outcome	• What is the optimal land-use in terms of the total monetary value of ecosystem services and other benefits provided?<br>• Designed for national policy making<br>• Provides policymakers with a spatially explicit land-allocation scenario that is optimised based on the market value of the considered ecosystem services	• While this type of model as well as our model, use optimisation to identify the most beneficial land conversions for a set of land-use benefits/ecosystem services, our model does not summarise different objectives in one metric<br>• While the focus of NEVO is to present one solution that is optimised based on the economic value, our model explores a broad range of potential priority weightings, which allows the analysis of trade-offs between the objectives and the exploration of a broad range of options
**Spatial Trade-Off Modelling** evaluate the land-use interdependencies between different objectives from an explicitly spatial perspective. Used in many contexts to identify optimal locations for specific land-uses and spatial trade-offs, a typical application considers ecosystem service trade-offs	• Spatially explicit evaluation of potential ecosystem services/ benefits in a location, usually for a range of benefits<br>• Explores the interaction between services and identifies trade-offs and synergies	• The focus is generally more on the local interactions between the services than on evaluating the trade-offs for policymaking on a national scale<br>• There are also some global studies on trade-offs between 2-3 objectives and most beneficial locations on a global scale	• What ecosystem services can be supplied in a location, and which trade-offs exist with other potential benefits?<br>• Exploring the spatially explicit potential for ecosystem service/benefit provision and potential conflicts, trade-offs or synergies between objectives	• Our model falls into this category<br>• While many existing trade-off studies are either on a global level or a regional level, we present a trade-off model that is suitable for national policy making<br>• We explore a broad range of options and analyse and summarise them in new ways

	• Can be used to identify optimal locations for certain land-uses or activities			

Supplementary Table 1 Overview of existing land-use models

Supplementary Table 2 Land conversions in the 231 scenarios

	Carbon	Prod.	Biodiv.	stays A	A -> P	A->PF	A->SnH	P->A	stays P	P->PF	P->SnH	PF->A	PF->P	stays PF	PF->SnH	SnH->A	SnH->P	SnH->PF	stays SnH
0-0-100	8770	62	349740	0.0%	0.0%	0.0%	25.8%	0.0%	0.0%	0.1%	31.5%	0.0%	0.0%	0.2%	6.3%	0.0%	0.0%	0.5%	35.7%
0-5-95	8911	330	349675	0.0%	0.0%	0.1%	25.7%	0.0%	0.0%	0.3%	31.3%	0.0%	0.0%	1.0%	5.5%	0.0%	0.0%	0.9%	35.3%
0-10-90	9087	680	349436	0.0%	0.0%	0.1%	25.6%	0.0%	0.0%	0.6%	31.0%	0.0%	0.0%	2.1%	4.4%	0.0%	0.0%	1.4%	34.8%
0-15-85	9165	903	349167	0.0%	0.0%	0.3%	25.5%	0.0%	0.0%	1.0%	30.6%	0.0%	0.0%	2.8%	3.7%	0.0%	0.0%	2.0%	34.2%
0-20-80	9227	1119	348774	0.0%	0.0%	0.5%	25.3%	0.0%	0.0%	1.6%	30.0%	0.0%	0.0%	3.4%	3.1%	0.0%	0.0%	2.7%	33.4%
0-25-75	9321	1392	348093	0.0%	0.0%	0.7%	25.1%	0.0%	0.0%	2.4%	29.2%	0.0%	0.0%	3.9%	2.6%	0.0%	0.1%	3.7%	32.4%
0-30-70	8839	2399	344726	0.0%	0.0%	0.8%	24.9%	0.0%	0.0%	3.3%	28.2%	0.0%	0.3%	4.3%	1.9%	0.0%	3.0%	4.5%	28.6%
0-35-65	7715	3983	338193	0.2%	0.0%	1.0%	24.5%	0.3%	0.3%	4.0%	26.9%	0.0%	0.8%	4.6%	1.1%	0.1%	7.5%	4.3%	24.3%
0-40-60	6062	6071	327364	1.0%	0.2%	1.1%	23.4%	1.3%	1.6%	3.8%	24.9%	0.0%	1.1%	4.7%	0.7%	0.3%	11.4%	3.1%	21.4%
0-45-55	3447	9863	303269	4.8%	0.4%	0.4%	20.1%	4.2%	3.6%	1.9%	21.9%	0.0%	1.3%	4.7%	0.5%	0.8%	15.3%	1.4%	18.7%
0-50-50	-3465	17518	242713	14.8%	1.7%	0.2%	9.1%	11.2%	8.5%	0.5%	11.3%	0.1%	1.5%	4.6%	0.3%	1.5%	18.3%	0.7%	15.7%
0-55-45	-10720	23525	188016	19.0%	6.5%	0.1%	0.1%	14.3%	16.6%	0.3%	0.4%	0.1%	1.9%	4.3%	0.2%	2.6%	20.5%	0.8%	12.1%
0-60-40	-13281	25077	170044	19.6%	6.0%	0.1%	0.1%	14.6%	16.3%	0.4%	0.3%	0.1%	2.2%	4.1%	0.2%	4.1%	25.1%	1.1%	5.9%
0-65-35	-13659	25291	167161	20.1%	5.6%	0.1%	0.1%	14.9%	16.1%	0.4%	0.3%	0.1%	2.4%	3.8%	0.1%	4.2%	25.7%	1.2%	5.0%
0-70-30	-13735	25338	166329	20.4%	5.3%	0.1%	0.0%	15.2%	15.9%	0.3%	0.2%	0.1%	2.6%	3.6%	0.1%	4.2%	25.7%	1.4%	4.8%
0-75-25	-13808	25375	165496	20.7%	5.0%	0.1%	0.0%	15.5%	15.7%	0.3%	0.1%	0.2%	2.8%	3.4%	0.1%	4.3%	25.6%	1.6%	4.5%

0-80-20	-13861	25396	164898	20.8%	4.9%	0.0%	0.0%	15.7%	15.6%	0.3%	0.1%	0.2%	2.9%	3.3%	0.1%	4.4%	25.6%	1.9%	4.2%
0-85-15	-13906	25408	164416	20.9%	4.8%	0.0%	0.0%	15.8%	15.5%	0.3%	0.0%	0.2%	3.0%	3.2%	0.1%	4.5%	25.6%	2.2%	3.9%
0-90-10	-13967	25417	163878	21.0%	4.8%	0.0%	0.0%	15.9%	15.4%	0.3%	0.0%	0.2%	3.1%	3.2%	0.1%	4.5%	25.5%	2.7%	3.4%
0-95-5	-14016	25422	163329	21.0%	4.7%	0.0%	0.0%	16.0%	15.3%	0.3%	0.0%	0.2%	3.1%	3.1%	0.1%	4.6%	25.5%	3.5%	2.6%
0-100-0	-14339	25424	159884	21.1%	4.7%	0.0%	0.0%	16.1%	15.3%	0.3%	0.0%	0.3%	3.2%	3.0%	0.0%	6.5%	25.5%	4.1%	0.0%
5-0-95	8816	117	349735	0.0%	0.0%	0.0%	25.8%	0.0%	0.0%	0.1%	31.5%	0.0%	0.0%	0.3%	6.2%	0.0%	0.0%	0.5%	35.7%
5-5-90	9002	448	349610	0.0%	0.0%	0.1%	25.7%	0.0%	0.0%	0.4%	31.2%	0.0%	0.0%	1.3%	5.2%	0.0%	0.0%	0.9%	35.3%
5-10-85	9140	749	349363	0.0%	0.0%	0.1%	25.6%	0.0%	0.0%	0.7%	30.9%	0.0%	0.0%	2.2%	4.3%	0.0%	0.0%	1.4%	34.7%
5-15-80	9210	962	349070	0.0%	0.0%	0.3%	25.5%	0.0%	0.0%	1.2%	30.4%	0.0%	0.0%	2.9%	3.6%	0.0%	0.0%	2.1%	34.0%
5-20-75	9299	1214	348555	0.0%	0.0%	0.5%	25.2%	0.0%	0.0%	1.9%	29.7%	0.0%	0.0%	3.5%	3.0%	0.0%	0.0%	3.0%	33.2%
5-25-70	9406	1503	347753	0.0%	0.0%	0.7%	25.1%	0.0%	0.0%	2.8%	28.8%	0.0%	0.0%	4.0%	2.4%	0.0%	0.1%	4.1%	32.0%
5-30-65	8957	2556	344111	0.0%	0.0%	0.9%	24.9%	0.1%	0.0%	3.7%	27.8%	0.0%	0.1%	4.4%	2.0%	0.0%	3.4%	4.9%	27.8%
5-35-60	7842	4134	337451	0.2%	0.0%	1.0%	24.6%	0.4%	0.2%	4.3%	26.6%	0.0%	0.6%	4.7%	1.2%	0.1%	7.9%	4.4%	23.7%
5-40-55	5881	6785	323148	1.8%	0.1%	1.0%	22.8%	1.9%	1.6%	3.8%	24.3%	0.0%	1.1%	4.8%	0.6%	0.4%	12.1%	3.0%	20.7%
5-45-50	2818	11112	294231	6.8%	0.2%	0.3%	18.4%	5.6%	3.6%	1.8%	20.6%	0.0%	1.2%	4.8%	0.4%	1.0%	15.7%	1.2%	18.2%
5-50-45	-5756	20238	218772	18.4%	3.5%	0.1%	3.8%	13.6%	11.0%	0.5%	6.5%	0.1%	1.5%	4.7%	0.2%	1.7%	18.0%	0.7%	15.7%
5-55-40	-10823	23855	184382	19.8%	5.8%	0.1%	0.1%	14.8%	16.1%	0.3%	0.4%	0.1%	1.7%	4.5%	0.2%	3.3%	20.9%	0.9%	10.9%
5-60-35	-13311	25217	168144	20.2%	5.4%	0.1%	0.1%	15.1%	15.8%	0.4%	0.3%	0.1%	2.0%	4.2%	0.2%	4.2%	25.5%	1.1%	5.3%
5-65-30	-13482	25308	166821	20.5%	5.1%	0.1%	0.0%	15.4%	15.6%	0.4%	0.2%	0.1%	2.3%	3.9%	0.2%	4.3%	25.6%	1.3%	5.0%
5-70-25	-13586	25354	165927	20.8%	4.9%	0.1%	0.0%	15.7%	15.5%	0.3%	0.2%	0.1%	2.5%	3.7%	0.1%	4.4%	25.6%	1.5%	4.7%
5-75-20	-13684	25383	165214	20.9%	4.8%	0.0%	0.0%	15.8%	15.4%	0.3%	0.1%	0.2%	2.7%	3.5%	0.1%	4.5%	25.5%	1.8%	4.4%
5-80-15	-13759	25401	164647	21.0%	4.7%	0.0%	0.0%	16.0%	15.3%	0.3%	0.0%	0.2%	2.8%	3.4%	0.1%	4.5%	25.5%	2.1%	4.0%
5-85-10	-13819	25410	164222	21.1%	4.7%	0.0%	0.0%	16.1%	15.2%	0.3%	0.0%	0.2%	2.9%	3.3%	0.1%	4.6%	25.5%	2.3%	3.8%
5-90-5	-13902	25419	163509	21.1%	4.7%	0.0%	0.0%	16.2%	15.1%	0.3%	0.0%	0.2%	3.0%	3.2%	0.1%	4.6%	25.5%	3.2%	2.8%

5-95-0	-13969	25422	162955	21.1%	4.6%	0.0%	0.0%	16.2%	15.1%	0.3%	0.0%	0.2%	3.1%	3.1%	0.1%	4.6%	25.5%	3.9%	2.1%
10-0-90	8875	196	349715	0.0%	0.0%	0.0%	25.8%	0.0%	0.0%	0.2%	31.4%	0.0%	0.0%	0.5%	6.0%	0.0%	0.0%	0.5%	35.6%
10-05-85	9073	548	349536	0.0%	0.0%	0.0%	25.7%	0.0%	0.0%	0.5%	31.1%	0.0%	0.0%	1.5%	5.0%	0.0%	0.0%	1.0%	35.2%
10-10-70	9184	811	349283	0.0%	0.0%	0.2%	25.6%	0.0%	0.0%	0.9%	30.7%	0.0%	0.0%	2.4%	4.1%	0.0%	0.0%	1.6%	34.6%
10-15-75	9259	1031	348936	0.0%	0.0%	0.3%	25.5%	0.0%	0.0%	1.4%	30.1%	0.0%	0.0%	3.0%	3.5%	0.0%	0.0%	2.3%	33.8%
10-20-70	9377	1321	348268	0.0%	0.0%	0.6%	25.2%	0.0%	0.0%	2.3%	29.3%	0.0%	0.0%	3.7%	2.8%	0.0%	0.0%	3.4%	32.8%
10-25-65	9484	1615	347361	0.0%	0.0%	0.7%	25.0%	0.0%	0.0%	3.3%	28.3%	0.0%	0.0%	4.2%	2.3%	0.0%	0.1%	4.6%	31.5%
10-30-60	8952	2755	343299	0.0%	0.0%	0.9%	24.9%	0.1%	0.0%	4.1%	27.4%	0.0%	0.0%	4.5%	1.9%	0.1%	3.9%	5.2%	27.0%
10-35-55	7940	4327	336396	0.2%	0.0%	1.0%	24.5%	0.5%	0.2%	4.4%	26.4%	0.0%	0.4%	4.8%	1.3%	0.2%	8.5%	4.5%	23.0%
10-40-50	5605	7705	317312	3.1%	0.1%	0.8%	21.8%	2.6%	1.5%	3.8%	23.7%	0.0%	1.0%	4.9%	0.6%	0.6%	13.0%	2.7%	19.9%
10-45-45	1774	12910	280303	9.9%	0.2%	0.2%	15.5%	7.6%	3.7%	1.7%	18.6%	0.0%	1.2%	4.9%	0.4%	1.2%	16.0%	1.1%	17.8%
10-50-40	-7052	21687	205205	19.9%	4.6%	0.1%	1.3%	14.8%	12.9%	0.5%	3.3%	0.1%	1.4%	4.7%	0.2%	2.0%	17.7%	0.8%	15.6%
10-55-35	-11044	24210	180126	20.4%	5.2%	0.1%	0.1%	15.4%	15.5%	0.3%	0.4%	0.1%	1.7%	4.5%	0.2%	3.9%	21.7%	1.0%	9.5%
10-60-30	-13216	25262	167358	20.7%	4.9%	0.1%	0.1%	15.6%	15.3%	0.4%	0.3%	0.1%	1.9%	4.3%	0.2%	4.4%	25.5%	1.2%	5.1%
10-65-25	-13345	25317	166493	20.9%	4.8%	0.1%	0.0%	15.8%	15.2%	0.4%	0.2%	0.1%	2.2%	4.0%	0.2%	4.4%	25.5%	1.3%	4.9%
10-70-20	-13476	25359	165645	21.0%	4.7%	0.0%	0.0%	16.0%	15.1%	0.3%	0.1%	0.2%	2.4%	3.8%	0.1%	4.5%	25.5%	1.6%	4.6%
10-75-15	-13573	25384	164979	21.1%	4.7%	0.0%	0.0%	16.2%	15.1%	0.3%	0.1%	0.2%	2.5%	3.7%	0.1%	4.5%	25.5%	2.0%	4.2%
10-80-10	-13657	25399	164482	21.1%	4.6%	0.0%	0.0%	16.3%	15.0%	0.3%	0.0%	0.2%	2.7%	3.5%	0.1%	4.6%	25.4%	2.2%	3.9%
10-85-5	-13735	25410	163895	21.2%	4.6%	0.0%	0.0%	16.3%	15.0%	0.3%	0.0%	0.2%	2.8%	3.4%	0.1%	4.6%	25.4%	2.8%	3.3%
10-90-0	-13826	25417	163287	21.2%	4.5%	0.0%	0.0%	16.4%	14.9%	0.3%	0.0%	0.2%	2.9%	3.3%	0.1%	4.7%	25.4%	3.5%	2.5%
15-0-85	8957	306	349667	0.0%	0.0%	0.0%	25.8%	0.0%	0.0%	0.3%	31.3%	0.0%	0.0%	0.8%	5.7%	0.0%	0.0%	0.5%	35.6%
15-5-80	9133	637	349454	0.0%	0.0%	0.0%	25.7%	0.0%	0.0%	0.7%	30.9%	0.0%	0.0%	1.8%	4.7%	0.0%	0.0%	1.0%	35.1%
15-10-75	9224	867	349194	0.0%	0.0%	0.2%	25.6%	0.0%	0.0%	1.1%	30.5%	0.0%	0.0%	2.5%	4.0%	0.0%	0.0%	1.7%	34.4%
15-15-70	9325	1129	348715	0.0%	0.0%	0.3%	25.4%	0.0%	0.0%	1.8%	29.7%	0.0%	0.0%	3.2%	3.3%	0.0%	0.0%	2.6%	33.5%

15-20-65	9464	1440	347902	0.0%	0.0%	0.6%	25.2%	0.0%	0.0%	2.8%	28.8%	0.0%	0.0%	3.8%	2.7%	0.0%	0.0%	3.8%	32.3%
15-25-60	9552	1721	346947	0.0%	0.0%	0.7%	25.0%	0.0%	0.0%	3.7%	27.9%	0.0%	0.0%	4.3%	2.2%	0.0%	0.1%	5.1%	31.0%
15-30-55	8897	2985	342323	0.1%	0.0%	0.9%	24.8%	0.1%	0.0%	4.3%	27.1%	0.0%	0.0%	4.6%	1.9%	0.1%	4.5%	5.5%	26.1%
15-35-50	7939	4694	334296	0.5%	0.0%	1.0%	24.3%	0.9%	0.3%	4.4%	26.0%	0.0%	0.1%	4.8%	1.5%	0.3%	9.1%	4.4%	22.4%
15-40-45	5259	8706	310470	4.4%	0.0%	0.5%	20.8%	3.6%	1.4%	3.7%	22.9%	0.0%	0.8%	4.9%	0.7%	0.8%	13.9%	2.4%	19.1%
15-45-40	44	15578	258207	14.8%	0.3%	0.2%	10.5%	10.8%	4.0%	1.7%	15.1%	0.1%	1.2%	4.9%	0.4%	1.4%	16.2%	1.0%	17.5%
15-50-35	-7636	22456	197252	20.6%	4.8%	0.1%	0.4%	15.6%	14.2%	0.5%	1.2%	0.1%	1.4%	4.8%	0.2%	2.6%	17.2%	0.9%	15.4%
15-55-30	-11280	24457	176936	20.8%	4.8%	0.1%	0.1%	15.9%	15.0%	0.3%	0.3%	0.1%	1.6%	4.6%	0.2%	4.2%	22.4%	1.1%	8.5%
15-60-25	-13113	25270	167011	21.0%	4.7%	0.1%	0.0%	16.0%	15.0%	0.4%	0.3%	0.1%	1.8%	4.4%	0.2%	4.5%	25.4%	1.2%	5.0%
15-65-20	-13236	25319	166201	21.1%	4.6%	0.0%	0.0%	16.2%	14.9%	0.3%	0.2%	0.1%	2.1%	4.1%	0.2%	4.5%	25.4%	1.4%	4.8%
15-70-15	-13371	25358	165375	21.2%	4.6%	0.0%	0.0%	16.4%	14.9%	0.3%	0.1%	0.2%	2.2%	3.9%	0.2%	4.6%	25.4%	1.8%	4.4%
15-75-10	-13474	25381	164778	21.2%	4.5%	0.0%	0.0%	16.4%	14.8%	0.3%	0.0%	0.2%	2.4%	3.8%	0.1%	4.6%	25.4%	2.1%	4.0%
15-80-5	-13557	25394	164256	21.3%	4.5%	0.0%	0.0%	16.5%	14.8%	0.3%	0.0%	0.2%	2.6%	3.6%	0.1%	4.7%	25.4%	2.6%	3.5%
15-85-0	-13647	25404	163664	21.3%	4.5%	0.0%	0.0%	16.6%	14.7%	0.3%	0.0%	0.2%	2.7%	3.5%	0.1%	4.7%	25.4%	3.1%	3.0%
20-0-80	9035	416	349600	0.0%	0.0%	0.0%	25.8%	0.0%	0.0%	0.5%	31.1%	0.0%	0.0%	1.1%	5.4%	0.0%	0.0%	0.6%	35.6%
20-5-75	9180	709	349371	0.0%	0.0%	0.0%	25.7%	0.0%	0.0%	0.9%	30.7%	0.0%	0.0%	2.0%	4.5%	0.0%	0.0%	1.1%	35.0%
20-10-70	9270	935	349068	0.0%	0.0%	0.2%	25.6%	0.0%	0.0%	1.5%	30.1%	0.0%	0.0%	2.6%	3.9%	0.0%	0.0%	1.9%	34.2%
20-15-65	9406	1252	348395	0.0%	0.0%	0.3%	25.4%	0.0%	0.0%	2.3%	29.3%	0.0%	0.0%	3.4%	3.1%	0.0%	0.0%	3.0%	33.1%
20-20-60	9541	1553	347505	0.0%	0.0%	0.6%	25.2%	0.0%	0.0%	3.3%	28.3%	0.0%	0.0%	4.0%	2.5%	0.0%	0.0%	4.3%	31.8%
20-25-55	9601	1802	346583	0.0%	0.0%	0.8%	25.0%	0.0%	0.0%	4.1%	27.5%	0.0%	0.0%	4.4%	2.1%	0.0%	0.1%	5.5%	30.5%
20-30-50	8819	3228	341244	0.1%	0.0%	0.9%	24.7%	0.2%	0.0%	4.4%	27.0%	0.0%	0.1%	4.6%	1.8%	0.1%	5.1%	5.7%	25.3%
20-35-45	7672	5460	329871	1.4%	0.0%	0.9%	23.5%	1.4%	0.4%	4.5%	25.3%	0.0%	0.1%	4.9%	1.5%	0.4%	9.8%	4.4%	21.6%
20-40-40	4802	9900	301625	6.3%	0.0%	0.4%	19.0%	4.9%	1.4%	3.5%	21.8%	0.0%	0.5%	5.0%	1.0%	1.0%	14.6%	2.2%	18.4%
20-45-35	-1735	17985	236964	18.8%	0.7%	0.2%	6.0%	13.7%	4.9%	1.7%	11.3%	0.1%	1.2%	4.9%	0.3%	1.7%	16.4%	1.0%	17.0%

20-50-30	-7963	22935	191690	21.0%	4.5%	0.1%	0.2%	16.2%	14.4%	0.5%	0.5%	0.1%	1.4%	4.8%	0.2%	3.3%	17.4%	1.0%	14.5%
20-55-25	-11523	24634	174552	21.1%	4.5%	0.1%	0.1%	16.3%	14.6%	0.4%	0.3%	0.1%	1.6%	4.6%	0.2%	4.4%	23.0%	1.1%	7.6%
20-60-20	-13030	25273	166686	21.2%	4.5%	0.1%	0.0%	16.4%	14.6%	0.3%	0.2%	0.1%	1.8%	4.4%	0.2%	4.6%	25.3%	1.3%	5.0%
20-65-15	-13145	25317	165888	21.3%	4.4%	0.0%	0.0%	16.6%	14.6%	0.3%	0.2%	0.1%	2.0%	4.2%	0.2%	4.6%	25.3%	1.5%	4.6%
20-70-10	-13274	25352	165094	21.3%	4.4%	0.0%	0.0%	16.7%	14.5%	0.3%	0.1%	0.2%	2.1%	4.0%	0.2%	4.7%	25.3%	1.9%	4.2%
20-75-5	-13368	25372	164501	21.4%	4.4%	0.0%	0.0%	16.8%	14.5%	0.3%	0.0%	0.2%	2.3%	3.9%	0.1%	4.8%	25.3%	2.5%	3.6%
20-80-0	-13461	25386	163915	21.4%	4.3%	0.0%	0.0%	16.8%	14.4%	0.3%	0.0%	0.2%	2.5%	3.7%	0.1%	4.8%	25.3%	3.0%	3.0%
25-0-75	9101	514	349522	0.0%	0.0%	0.0%	25.8%	0.0%	0.0%	0.7%	30.9%	0.0%	0.0%	1.4%	5.1%	0.0%	0.0%	0.6%	35.5%
25-5-70	9224	774	349277	0.0%	0.0%	0.0%	25.7%	0.0%	0.0%	1.2%	30.4%	0.0%	0.0%	2.1%	4.4%	0.0%	0.0%	1.3%	34.9%
25-10-65	9333	1030	348862	0.0%	0.0%	0.2%	25.6%	0.0%	0.0%	1.9%	29.7%	0.0%	0.0%	2.8%	3.7%	0.0%	0.0%	2.2%	33.9%
25-15-60	9493	1384	347997	0.0%	0.0%	0.4%	25.4%	0.0%	0.0%	2.9%	28.7%	0.0%	0.0%	3.6%	2.9%	0.0%	0.0%	3.5%	32.7%
25-20-55	9606	1655	347099	0.0%	0.0%	0.6%	25.1%	0.0%	0.0%	3.8%	27.8%	0.0%	0.0%	4.1%	2.4%	0.0%	0.0%	4.8%	31.3%
25-25-50	9630	1872	346252	0.0%	0.0%	0.8%	24.9%	0.0%	0.0%	4.2%	27.3%	0.0%	0.0%	4.5%	2.0%	0.0%	0.1%	5.8%	30.2%
25-30-45	8727	3506	339916	0.2%	0.0%	0.9%	24.7%	0.3%	0.0%	4.5%	26.7%	0.0%	0.1%	4.7%	1.7%	0.2%	5.7%	5.9%	24.5%
25-35-40	7264	6431	323908	2.6%	0.0%	0.5%	22.6%	2.1%	0.5%	4.4%	24.6%	0.0%	0.1%	4.9%	1.5%	0.6%	10.6%	4.2%	20.7%
25-40-35	3878	11809	286422	9.7%	0.1%	0.4%	15.6%	7.1%	1.4%	3.3%	19.8%	0.0%	0.2%	4.9%	1.3%	1.2%	15.0%	2.1%	17.8%
25-45-30	-2773	19245	225055	20.4%	1.1%	0.2%	4.1%	15.2%	5.8%	1.9%	8.7%	0.1%	1.1%	4.9%	0.4%	2.2%	16.5%	1.0%	16.4%
25-50-25	-8121	23164	188675	21.2%	4.3%	0.1%	0.2%	16.7%	13.9%	0.6%	0.4%	0.1%	1.4%	4.8%	0.2%	3.8%	17.8%	1.1%	13.5%
25-55-20	-11709	24765	172577	21.3%	4.3%	0.1%	0.1%	16.8%	14.1%	0.4%	0.3%	0.1%	1.5%	4.6%	0.2%	4.6%	23.4%	1.2%	7.0%
25-60-15	-12943	25271	166303	21.4%	4.3%	0.0%	0.0%	17.0%	14.1%	0.3%	0.2%	0.1%	1.7%	4.5%	0.2%	4.7%	25.2%	1.4%	4.8%
25-65-10	-13056	25310	165529	21.5%	4.3%	0.0%	0.0%	17.1%	14.1%	0.3%	0.1%	0.1%	1.9%	4.3%	0.2%	4.8%	25.2%	1.7%	4.4%
25-70-5	-13177	25341	164798	21.5%	4.2%	0.0%	0.0%	17.1%	14.1%	0.3%	0.0%	0.2%	2.1%	4.1%	0.2%	4.8%	25.2%	2.2%	3.9%
25-75-0	-13279	25360	164154	21.5%	4.2%	0.0%	0.0%	17.1%	14.1%	0.3%	0.0%	0.2%	2.2%	4.0%	0.2%	4.9%	25.2%	2.9%	3.1%
30-0-70	9158	602	349433	0.0%	0.0%	0.0%	25.8%	0.0%	0.0%	0.9%	30.7%	0.0%	0.0%	1.6%	4.9%	0.0%	0.0%	0.7%	35.4%

30-5-65	9274	844	349149	0.0%	0.0%	0.0%	25.7%	0.0%	0.0%	1.6%	30.0%	0.0%	0.0%	2.2%	4.2%	0.0%	0.0%	1.5%	34.6%
30-10-60	9418	1164	348524	0.0%	0.0%	0.2%	25.6%	0.0%	0.0%	2.5%	29.1%	0.0%	0.0%	3.0%	3.5%	0.0%	0.0%	2.6%	33.5%
30-15-55	9569	1505	347579	0.0%	0.0%	0.4%	25.4%	0.0%	0.0%	3.4%	28.2%	0.0%	0.0%	3.8%	2.7%	0.0%	0.0%	4.0%	32.1%
30-20-50	9649	1734	346752	0.0%	0.0%	0.7%	25.1%	0.0%	0.0%	4.1%	27.5%	0.0%	0.0%	4.2%	2.2%	0.0%	0.0%	5.3%	30.9%
30-25-45	9647	1953	345843	0.0%	0.0%	0.9%	24.9%	0.1%	0.0%	4.4%	27.2%	0.0%	0.0%	4.6%	1.9%	0.1%	0.1%	6.2%	29.8%
30-30-40	8601	3916	337762	0.5%	0.0%	0.9%	24.4%	0.6%	0.0%	4.6%	26.4%	0.0%	0.1%	4.8%	1.6%	0.3%	6.3%	6.0%	23.6%
30-35-35	6716	7590	316282	4.0%	0.0%	0.4%	21.4%	3.1%	0.6%	4.2%	23.7%	0.0%	0.1%	4.9%	1.5%	0.9%	11.7%	4.0%	19.6%
30-40-30	2262	14275	266008	14.3%	0.1%	0.3%	11.1%	10.1%	1.6%	3.1%	16.9%	0.0%	0.2%	4.9%	1.3%	1.5%	15.2%	2.0%	17.4%
30-45-25	-3653	20253	214515	20.9%	1.6%	0.2%	3.0%	16.5%	6.9%	2.2%	6.0%	0.1%	0.8%	4.9%	0.7%	2.7%	16.9%	1.1%	15.4%
30-50-20	-8195	23290	186677	21.5%	4.1%	0.1%	0.1%	17.3%	13.3%	0.7%	0.3%	0.1%	1.4%	4.8%	0.2%	4.2%	18.0%	1.1%	12.8%
30-55-15	-11830	24853	170970	21.6%	4.1%	0.1%	0.0%	17.4%	13.6%	0.4%	0.2%	0.1%	1.5%	4.7%	0.2%	4.8%	23.6%	1.4%	6.4%
30-60-10	-12865	25262	165945	21.6%	4.1%	0.0%	0.0%	17.4%	13.7%	0.3%	0.2%	0.1%	1.7%	4.5%	0.2%	4.9%	25.1%	1.6%	4.6%
30-65-5	-12982	25300	165160	21.6%	4.1%	0.0%	0.0%	17.5%	13.8%	0.3%	0.1%	0.2%	1.9%	4.3%	0.2%	4.9%	25.1%	2.1%	4.1%
30-70-0	-13089	25326	164576	21.6%	4.1%	0.0%	0.0%	17.5%	13.8%	0.3%	0.1%	0.2%	2.0%	4.2%	0.2%	4.9%	25.1%	2.4%	3.7%
35-0-65	9212	684	349329	0.0%	0.0%	0.0%	25.8%	0.0%	0.0%	1.2%	30.4%	0.0%	0.0%	1.8%	4.7%	0.0%	0.0%	0.9%	35.3%
35-5-60	9339	936	348948	0.0%	0.0%	0.0%	25.7%	0.0%	0.0%	2.1%	29.5%	0.0%	0.0%	2.4%	4.1%	0.0%	0.0%	1.8%	34.3%
35-10-55	9509	1310	348098	0.0%	0.0%	0.2%	25.6%	0.0%	0.0%	3.1%	28.5%	0.0%	0.0%	3.3%	3.2%	0.0%	0.0%	3.2%	33.0%
35-15-50	9628	1606	347184	0.0%	0.0%	0.4%	25.4%	0.0%	0.0%	3.9%	27.7%	0.0%	0.0%	3.9%	2.5%	0.0%	0.0%	4.5%	31.6%
35-20-45	9676	1794	346457	0.0%	0.0%	0.7%	25.1%	0.0%	0.0%	4.3%	27.3%	0.0%	0.0%	4.4%	2.1%	0.0%	0.0%	5.7%	30.5%
35-25-40	9658	2048	345336	0.1%	0.0%	0.9%	24.8%	0.1%	0.0%	4.5%	27.0%	0.0%	0.0%	4.7%	1.8%	0.1%	0.1%	6.6%	29.4%
35-30-35	8383	4610	333778	1.5%	0.0%	0.6%	23.7%	1.0%	0.1%	4.7%	25.8%	0.0%	0.1%	4.8%	1.6%	0.5%	6.9%	6.1%	22.7%
35-35-30	5981	9060	305707	6.1%	0.0%	0.3%	19.4%	4.5%	0.7%	4.0%	22.5%	0.0%	0.2%	4.9%	1.4%	1.1%	12.8%	3.8%	18.5%
35-40-25	435	16675	245057	18.3%	0.3%	0.3%	6.9%	13.2%	2.0%	3.0%	13.4%	0.1%	0.2%	4.9%	1.3%	1.9%	15.5%	1.9%	16.8%
35-45-20	-4625	21201	204098	21.4%	2.3%	0.3%	1.8%	17.5%	8.3%	2.4%	3.3%	0.1%	0.3%	4.9%	1.2%	3.2%	17.3%	1.2%	14.5%

35-50-15	-8205	23353	185202	21.7%	3.8%	0.1%	0.1%	17.9%	12.5%	0.8%	0.3%	0.1%	1.3%	4.8%	0.2%	4.5%	18.1%	1.3%	12.2%
35-55-10	-11895	24907	169658	21.8%	3.9%	0.1%	0.0%	17.9%	13.0%	0.4%	0.2%	0.1%	1.5%	4.7%	0.2%	4.9%	23.8%	1.6%	5.9%
35-60-5	-12781	25247	165512	21.8%	3.9%	0.0%	0.0%	17.9%	13.3%	0.3%	0.1%	0.1%	1.7%	4.5%	0.2%	5.0%	25.0%	2.0%	4.2%
35-65-0	-12906	25283	164855	21.8%	3.9%	0.0%	0.0%	17.9%	13.4%	0.3%	0.1%	0.2%	1.8%	4.3%	0.2%	5.0%	25.0%	2.3%	3.8%
40-0-60	9270	766	349185	0.0%	0.0%	0.0%	25.8%	0.0%	0.0%	1.7%	29.9%	0.0%	0.0%	1.9%	4.5%	0.0%	0.0%	1.1%	35.1%
40-5-55	9427	1062	348619	0.0%	0.0%	0.0%	25.7%	0.0%	0.0%	2.7%	28.9%	0.0%	0.0%	2.6%	3.9%	0.0%	0.0%	2.3%	33.8%
40-10-50	9588	1443	347655	0.0%	0.0%	0.2%	25.6%	0.0%	0.0%	3.6%	28.0%	0.0%	0.0%	3.5%	3.0%	0.0%	0.0%	3.7%	32.4%
40-15-45	9669	1688	346833	0.0%	0.0%	0.4%	25.3%	0.0%	0.0%	4.2%	27.4%	0.0%	0.0%	4.1%	2.4%	0.0%	0.0%	5.0%	31.1%
40-20-40	9700	1857	346118	0.0%	0.0%	0.8%	25.0%	0.0%	0.0%	4.5%	27.1%	0.0%	0.0%	4.5%	2.0%	0.0%	0.0%	6.1%	30.1%
40-25-35	9652	2184	344582	0.2%	0.0%	0.9%	24.7%	0.2%	0.0%	4.7%	26.7%	0.0%	0.0%	4.7%	1.8%	0.1%	0.2%	7.0%	28.8%
40-30-30	8039	5459	328680	2.5%	0.0%	0.3%	22.9%	1.7%	0.1%	4.6%	25.2%	0.0%	0.1%	4.8%	1.6%	0.7%	7.6%	6.0%	21.8%
40-35-25	4880	11028	290367	9.4%	0.0%	0.3%	16.1%	6.6%	0.7%	3.8%	20.5%	0.0%	0.2%	4.9%	1.4%	1.3%	13.5%	3.6%	17.7%
40-40-20	-750	18069	232010	20.0%	0.7%	0.2%	4.8%	15.1%	2.6%	3.1%	10.8%	0.1%	0.2%	4.9%	1.3%	2.5%	15.8%	1.9%	16.0%
40-45-15	-5553	21918	196490	21.7%	2.8%	0.3%	1.0%	18.5%	9.1%	2.2%	1.8%	0.1%	0.3%	4.9%	1.2%	3.8%	17.4%	1.3%	13.7%
40-50-10	-8038	23323	184172	22.0%	3.5%	0.1%	0.1%	18.6%	11.5%	0.9%	0.5%	0.1%	1.2%	4.8%	0.3%	4.8%	18.2%	1.4%	11.7%
40-55-5	-11824	24897	168818	22.0%	3.6%	0.1%	0.0%	18.6%	12.4%	0.5%	0.1%	0.1%	1.5%	4.7%	0.2%	5.1%	23.7%	2.0%	5.3%
40-60-0	-12675	25217	165177	22.0%	3.7%	0.0%	0.0%	18.5%	12.7%	0.3%	0.1%	0.1%	1.6%	4.5%	0.2%	5.2%	24.8%	2.3%	3.9%
45-0-55	9341	863	348971	0.0%	0.0%	0.0%	25.8%	0.0%	0.0%	2.2%	29.4%	0.0%	0.0%	2.1%	4.4%	0.0%	0.0%	1.5%	34.7%
45-5-50	9517	1203	348205	0.0%	0.0%	0.0%	25.7%	0.0%	0.0%	3.3%	28.3%	0.0%	0.0%	2.8%	3.7%	0.0%	0.0%	2.8%	33.3%
45-10-45	9642	1544	347274	0.0%	0.0%	0.2%	25.5%	0.0%	0.0%	4.0%	27.6%	0.0%	0.0%	3.7%	2.8%	0.0%	0.0%	4.2%	31.9%
45-15-40	9698	1751	346526	0.0%	0.0%	0.5%	25.3%	0.0%	0.0%	4.4%	27.2%	0.0%	0.0%	4.2%	2.3%	0.0%	0.0%	5.5%	30.7%
45-20-35	9718	1925	345728	0.0%	0.0%	0.8%	24.9%	0.0%	0.0%	4.6%	26.9%	0.0%	0.0%	4.6%	1.9%	0.0%	0.0%	6.5%	29.6%
45-25-30	9592	2525	342636	0.7%	0.0%	0.8%	24.3%	0.5%	0.0%	4.7%	26.4%	0.0%	0.0%	4.8%	1.7%	0.3%	0.3%	7.5%	28.1%
45-30-25	7483	6702	320572	4.1%	0.0%	0.2%	21.4%	2.7%	0.1%	4.4%	24.3%	0.0%	0.1%	4.8%	1.5%	1.0%	8.6%	5.8%	20.7%

45-35-20	3314	13451	270156	13.8%	0.1%	0.2%	11.7%	9.4%	0.7%	3.7%	17.8%	0.0%	0.2%	4.9%	1.4%	1.7%	13.8%	3.6%	17.0%
45-40-15	-1583	18956	222956	20.7%	0.8%	0.2%	4.1%	16.8%	2.8%	3.6%	8.4%	0.1%	0.3%	4.9%	1.3%	2.9%	16.2%	2.0%	15.0%
45-45-10	-6032	22280	192021	22.0%	3.0%	0.2%	0.6%	19.5%	9.0%	1.9%	1.3%	0.1%	0.3%	4.9%	1.2%	4.3%	17.5%	1.4%	13.0%
45-50-5	-7439	23089	183772	22.3%	3.3%	0.1%	0.1%	19.4%	10.4%	0.9%	0.9%	0.1%	0.4%	4.8%	1.2%	5.1%	18.4%	1.8%	10.9%
45-55-0	-9781	24111	174516	22.3%	3.4%	0.1%	0.0%	19.3%	11.6%	0.6%	0.1%	0.1%	1.5%	4.7%	0.2%	5.3%	20.4%	2.3%	8.1%
50-0-50	9428	981	348648	0.0%	0.0%	0.0%	25.8%	0.0%	0.0%	2.9%	28.7%	0.0%	0.0%	2.3%	4.2%	0.0%	0.0%	1.9%	34.2%
50-5-45	9596	1340	347760	0.0%	0.0%	0.0%	25.7%	0.0%	0.0%	3.8%	27.8%	0.0%	0.0%	3.1%	3.4%	0.0%	0.0%	3.4%	32.7%
50-10-40	9683	1631	346917	0.0%	0.0%	0.3%	25.5%	0.0%	0.0%	4.3%	27.3%	0.0%	0.0%	3.9%	2.6%	0.0%	0.0%	4.8%	31.4%
50-15-35	9725	1809	346191	0.0%	0.0%	0.5%	25.3%	0.0%	0.0%	4.6%	27.0%	0.0%	0.0%	4.4%	2.1%	0.0%	0.0%	6.0%	30.1%
50-20-30	9728	2007	345235	0.1%	0.0%	0.9%	24.8%	0.1%	0.0%	4.8%	26.8%	0.0%	0.0%	4.7%	1.8%	0.1%	0.0%	7.1%	29.0%
50-25-25	9451	3099	339192	1.7%	0.0%	0.4%	23.6%	0.8%	0.0%	4.7%	26.1%	0.0%	0.0%	4.8%	1.7%	0.5%	0.5%	7.7%	27.5%
50-30-20	6624	8441	308093	6.4%	0.0%	0.2%	19.2%	4.3%	0.2%	4.2%	22.9%	0.0%	0.1%	4.8%	1.5%	1.2%	10.2%	5.8%	18.9%
50-35-15	1671	15629	251217	17.4%	0.1%	0.2%	8.1%	12.5%	0.8%	3.7%	14.7%	0.1%	0.2%	4.9%	1.4%	2.2%	14.0%	3.6%	16.3%
50-40-10	-2422	19742	214450	21.3%	0.9%	0.2%	3.4%	18.3%	3.2%	4.7%	5.4%	0.1%	0.3%	4.9%	1.3%	3.4%	16.4%	1.9%	14.5%
50-45-5	-6225	22431	189192	22.5%	2.6%	0.2%	0.4%	20.3%	8.6%	1.5%	1.1%	0.1%	0.3%	4.8%	1.2%	4.7%	17.5%	1.7%	12.2%
50-50-0	-7465	23110	182279	22.6%	2.9%	0.1%	0.1%	20.2%	9.7%	0.9%	0.8%	0.1%	0.4%	4.8%	1.2%	5.3%	18.5%	2.1%	10.2%
55-0-45	9515	1111	348256	0.0%	0.0%	0.0%	25.8%	0.0%	0.0%	3.5%	28.1%	0.0%	0.0%	2.4%	4.0%	0.0%	0.0%	2.5%	33.7%
55-5-40	9651	1449	347379	0.0%	0.0%	0.0%	25.7%	0.0%	0.0%	4.2%	27.4%	0.0%	0.0%	3.3%	3.2%	0.0%	0.0%	4.0%	32.2%
55-10-35	9716	1704	346563	0.0%	0.0%	0.3%	25.5%	0.0%	0.0%	4.5%	27.1%	0.0%	0.0%	4.0%	2.5%	0.0%	0.0%	5.3%	30.8%
55-15-30	9747	1861	345841	0.0%	0.0%	0.5%	25.2%	0.0%	0.0%	4.8%	26.8%	0.0%	0.0%	4.5%	2.0%	0.0%	0.0%	6.6%	29.5%
55-20-25	9721	2128	344533	0.2%	0.0%	0.9%	24.7%	0.1%	0.0%	4.8%	26.6%	0.0%	0.0%	4.7%	1.8%	0.1%	0.0%	7.5%	28.5%
55-25-20	9189	3835	334610	2.7%	0.0%	0.2%	22.8%	1.3%	0.0%	4.7%	25.6%	0.0%	0.0%	4.8%	1.7%	0.8%	1.0%	7.9%	26.5%
55-30-15	5479	10511	291710	9.5%	0.0%	0.2%	16.0%	6.2%	0.3%	4.1%	21.0%	0.0%	0.1%	4.9%	1.5%	1.6%	11.5%	5.9%	17.1%
55-35-10	234	17306	235731	19.6%	0.4%	0.2%	5.6%	14.9%	1.3%	3.7%	11.6%	0.1%	0.2%	4.9%	1.3%	2.8%	14.4%	3.5%	15.4%

55-40-5	-3156	20360	207280	22.0%	1.0%	0.2%	2.6%	19.8%	3.3%	4.7%	3.7%	0.1%	0.3%	4.8%	1.3%	3.9%	16.5%	2.0%	13.7%
55-45-0	-6338	22513	186544	23.3%	1.9%	0.3%	0.3%	21.4%	8.0%	1.2%	1.0%	0.1%	0.3%	4.8%	1.2%	5.1%	17.5%	2.1%	11.4%
60-0-40	9590	1240	347846	0.0%	0.0%	0.0%	25.8%	0.0%	0.0%	4.0%	27.6%	0.0%	0.0%	2.6%	3.8%	0.0%	0.0%	3.1%	33.1%
60-5-35	9693	1541	347009	0.0%	0.0%	0.0%	25.7%	0.0%	0.0%	4.5%	27.1%	0.0%	0.0%	3.5%	3.0%	0.0%	0.0%	4.6%	31.6%
60-10-30	9744	1770	346187	0.0%	0.0%	0.3%	25.4%	0.0%	0.0%	4.7%	26.9%	0.0%	0.0%	4.2%	2.3%	0.0%	0.0%	6.0%	30.1%
60-15-25	9758	1906	345533	0.0%	0.0%	0.6%	25.1%	0.0%	0.0%	4.9%	26.7%	0.0%	0.0%	4.6%	1.9%	0.0%	0.0%	7.2%	28.9%
60-20-20	9655	2556	341918	1.1%	0.0%	0.6%	24.1%	0.4%	0.0%	4.9%	26.4%	0.0%	0.0%	4.7%	1.7%	0.3%	0.0%	7.8%	28.0%
60-25-15	8606	5257	324997	4.6%	0.0%	0.2%	21.0%	2.5%	0.0%	4.6%	24.6%	0.0%	0.0%	4.8%	1.6%	1.1%	2.2%	8.5%	24.4%
60-30-10	4052	12692	273771	13.4%	0.0%	0.1%	12.2%	8.6%	0.5%	4.0%	18.5%	0.1%	0.1%	4.9%	1.4%	2.0%	11.9%	5.8%	16.4%
60-35-5	-641	18226	226494	20.6%	0.5%	0.1%	4.5%	16.8%	1.5%	4.1%	9.2%	0.1%	0.2%	4.9%	1.3%	3.3%	14.7%	3.5%	14.7%
60-40-0	-3840	20878	200694	22.7%	1.1%	0.2%	1.8%	21.1%	3.4%	4.2%	2.8%	0.1%	0.3%	4.8%	1.3%	4.5%	16.5%	2.3%	12.8%
65-0-35	9645	1350	347474	0.0%	0.0%	0.0%	25.8%	0.0%	0.0%	4.4%	27.2%	0.0%	0.0%	2.9%	3.6%	0.0%	0.0%	3.6%	32.5%
65-5-30	9729	1627	346617	0.0%	0.0%	0.1%	25.7%	0.0%	0.0%	4.7%	26.9%	0.0%	0.0%	3.7%	2.8%	0.0%	0.0%	5.2%	30.9%
65-10-25	9764	1821	345841	0.0%	0.0%	0.4%	25.4%	0.0%	0.0%	4.9%	26.7%	0.0%	0.0%	4.3%	2.2%	0.0%	0.0%	6.8%	29.4%
65-15-20	9758	1982	345101	0.1%	0.0%	0.7%	25.0%	0.0%	0.0%	5.0%	26.6%	0.0%	0.0%	4.6%	1.9%	0.0%	0.0%	7.6%	28.5%
65-20-15	9568	3013	338714	1.9%	0.0%	0.3%	23.5%	0.7%	0.0%	4.8%	26.1%	0.0%	0.0%	4.8%	1.7%	0.5%	0.0%	8.5%	27.2%
65-25-10	7711	7016	313079	6.7%	0.0%	0.1%	19.0%	3.9%	0.0%	4.5%	23.2%	0.0%	0.0%	4.8%	1.6%	1.4%	4.2%	9.0%	21.5%
65-30-5	2456	14803	255745	16.9%	0.0%	0.1%	8.7%	11.7%	0.5%	3.9%	15.5%	0.1%	0.2%	4.9%	1.4%	2.6%	12.2%	5.6%	15.8%
65-35-0	-1431	18970	218122	21.4%	0.5%	0.1%	3.8%	18.6%	1.5%	5.2%	6.3%	0.1%	0.2%	4.8%	1.3%	3.7%	14.9%	3.6%	14.0%
70-0-30	9691	1451	347084	0.0%	0.0%	0.0%	25.8%	0.0%	0.0%	4.6%	27.0%	0.0%	0.0%	3.1%	3.4%	0.0%	0.0%	4.3%	31.8%
70-5-25	9759	1703	346200	0.0%	0.0%	0.1%	25.7%	0.0%	0.0%	4.8%	26.8%	0.0%	0.0%	3.9%	2.6%	0.0%	0.0%	6.0%	30.1%
70-10-20	9774	1855	345592	0.0%	0.0%	0.4%	25.4%	0.0%	0.0%	5.0%	26.6%	0.0%	0.0%	4.4%	2.1%	0.0%	0.0%	7.2%	28.9%
70-15-15	9748	2134	344110	0.3%	0.0%	0.7%	24.8%	0.1%	0.0%	5.0%	26.5%	0.0%	0.0%	4.7%	1.8%	0.1%	0.0%	8.1%	27.9%
70-20-10	9345	3858	331962	3.5%	0.0%	0.2%	22.1%	1.3%	0.0%	4.8%	25.5%	0.0%	0.0%	4.8%	1.7%	0.9%	0.0%	9.3%	26.0%

70-25-5	6197	9597	294731	9.7%	0.0%	0.1%	15.9%	6.0%	0.0%	4.4%	21.2%	0.0%	0.1%	4.9%	1.5%	1.8%	7.9%	9.1%	17.4%
70-30-0	927	16612	238936	19.6%	0.0%	0.1%	6.0%	14.8%	0.6%	4.0%	12.2%	0.1%	0.2%	4.9%	1.4%	3.1%	12.6%	5.5%	14.9%
75-0-25	9732	1547	346648	0.0%	0.0%	0.0%	25.8%	0.0%	0.0%	4.8%	26.8%	0.0%	0.0%	3.3%	3.2%	0.0%	0.0%	5.2%	31.0%
75-5-20	9777	1760	345855	0.0%	0.0%	0.1%	25.7%	0.0%	0.0%	5.0%	26.6%	0.0%	0.0%	4.1%	2.4%	0.0%	0.0%	6.7%	29.4%
75-10-15	9781	1886	345330	0.0%	0.0%	0.5%	25.3%	0.0%	0.0%	5.1%	26.5%	0.0%	0.0%	4.5%	2.0%	0.0%	0.0%	7.7%	28.4%
75-15-10	9679	2685	340113	1.4%	0.0%	0.4%	23.9%	0.4%	0.0%	5.0%	26.2%	0.0%	0.0%	4.7%	1.8%	0.3%	0.0%	9.2%	26.6%
75-20-5	9047	4657	325528	4.7%	0.0%	0.1%	20.9%	2.1%	0.0%	4.9%	24.6%	0.0%	0.0%	4.8%	1.7%	1.2%	0.0%	10.6%	24.3%
75-25-0	4838	11677	277977	13.1%	0.0%	0.1%	12.6%	8.0%	0.1%	4.5%	19.0%	0.1%	0.1%	4.9%	1.5%	2.3%	9.1%	9.0%	15.7%
80-0-20	9762	1628	346222	0.0%	0.0%	0.0%	25.8%	0.0%	0.0%	5.0%	26.6%	0.0%	0.0%	3.6%	2.9%	0.0%	0.0%	6.0%	30.1%
80-5-15	9785	1797	345612	0.0%	0.0%	0.2%	25.6%	0.0%	0.0%	5.1%	26.5%	0.0%	0.0%	4.2%	2.3%	0.0%	0.0%	7.2%	28.9%
80-10-10	9797	1986	344181	0.1%	0.0%	0.6%	25.2%	0.0%	0.0%	5.1%	26.4%	0.0%	0.0%	4.5%	2.0%	0.0%	0.0%	8.8%	27.3%
80-15-5	9535	3297	335464	2.6%	0.0%	0.2%	23.0%	0.8%	0.0%	5.1%	25.7%	0.0%	0.0%	4.7%	1.8%	0.6%	0.0%	10.5%	25.0%
80-20-0	8410	5980	314982	7.0%	0.0%	0.1%	18.7%	3.6%	0.0%	4.8%	23.3%	0.0%	0.0%	4.8%	1.6%	1.6%	0.0%	12.0%	22.5%
85-0-15	9779	1689	345910	0.0%	0.0%	0.0%	25.8%	0.0%	0.0%	5.0%	26.5%	0.0%	0.0%	3.8%	2.7%	0.0%	0.0%	6.6%	29.6%
85-5-10	9801	1853	344955	0.0%	0.0%	0.2%	25.5%	0.0%	0.0%	5.1%	26.5%	0.0%	0.0%	4.3%	2.2%	0.0%	0.0%	8.2%	28.0%
85-10-5	9774	2236	342258	0.5%	0.0%	0.5%	24.8%	0.1%	0.0%	5.2%	26.3%	0.0%	0.0%	4.5%	1.9%	0.2%	0.0%	10.3%	25.7%
85-15-0	9302	4057	328744	4.0%	0.0%	0.1%	21.6%	1.3%	0.0%	5.2%	25.0%	0.0%	0.0%	4.7%	1.7%	1.0%	0.0%	12.2%	22.9%
90-0-10	9793	1742	345472	0.0%	0.0%	0.0%	25.7%	0.0%	0.0%	5.1%	26.5%	0.0%	0.0%	3.9%	2.6%	0.0%	0.0%	7.4%	28.7%
90-5-5	9818	1922	343797	0.0%	0.0%	0.3%	25.4%	0.0%	0.0%	5.2%	26.3%	0.0%	0.0%	4.3%	2.2%	0.0%	0.0%	9.9%	26.2%
90-10-0	9679	2764	338343	1.7%	0.0%	0.2%	23.8%	0.3%	0.0%	5.3%	25.9%	0.0%	0.0%	4.6%	1.9%	0.3%	0.0%	12.2%	23.6%
95-0-5	9818	1826	344212	0.0%	0.0%	0.1%	25.7%	0.0%	0.0%	5.2%	26.3%	0.0%	0.0%	4.0%	2.5%	0.0%	0.0%	9.2%	27.0%
95-5-0	9820	1957	342923	0.0%	0.0%	0.4%	25.4%	0.0%	0.0%	5.4%	26.2%	0.0%	0.0%	4.4%	2.1%	0.0%	0.0%	12.1%	24.1%
100-0-0	9825	1874	343210	0.0%	0.0%	0.1%	25.6%	0.0%	0.0%	5.4%	26.2%	0.0%	0.0%	4.1%	2.4%	0.0%	0.0%	11.7%	24.5%

Supplementary Table 2 Land conversion in the 231 scenarios

Supplementary Table 3 Distance from the current land-use performance to the Pareto frontier

		Current	Distance to Pareto frontier	Percentage Improvement	Synergies	
Carbon	Normalised	0.36	0.30	+128.9%	with Biodiversity	0.12
	Mio. t CO_2-equ·yr^{-1}	-55.76	71.9			+22,530 Biodiv. Indic. points
Production	Normalised	0.62	0.15	23.6%	None	-
	Billion £· yr^{-1}	15.8	3.74			-
Biodiversity	Normalised	0.34	0.17	14.2%	with Carbon sequ.	0.16
	Biodiv. Indic. points	225,000	31,920			37.9 mio t CO_2-equ·yr^{-1}

Supplementary Table 3 Distance from the current land performance to the Pareto frontier along each axis as a measure of the inefficiency of current land-use

Supplementary Table 4 Carbon sequestration modelling – Livestock assumptions

	LSU per pasture area (mean 2016 – 2020)	LSU coefficients	Livestock density	Type share	Livestock density for sub-group	Emissions per animal	Emissions per ha pasture
	[LSU/ha]	[LSU/animal]	[animals/ha]	[%]	[anim./ha]	[CO_2-equ./animal]	[CO_2-equ./ha]
Cattle	0.7845	0.8	0.9806	66% Beef cows	0.6481	6.4431	4.1756
				34% Dairy cows	0.3325	4.1687	1.3863
Sheep	0.2984	0.1	2.9842		2.9842	0.6894	2.0571

Supplementary Table 4 Carbon sequestration modelling - Livestock assumptions

Supplementary Table 5 Carbon sequestration modelling – Forest assumptions

	Broadleaved forest	Coniferous forest
Age distribution	•Existing forests baseline: Based on the age distribution of broadleaved forests in the Forestry Statistics 2022[1]<br>•Existing forest in 2050: Based on the age distribution of the broadleaved forest baseline and shifted by 30 years<br>•New planted forests: Consistent planting from 2020-2050	•Existing forests baseline: Based on the age distribution of coniferous forests in the Forestry Statistics 2022 distribution of coniferous forests in the Forestry Statistics 2022[1]<br>•Existing forest in 2050: Based on the age distribution of coniferous forests in the Forestry Statistics 2023 with felling and replanting at the age of maximum mean annual volume increment[2]<br>•New planted forests: Consistent planting from 2020-2050

Species composition	•Existing forests baseline & 2050: Based on the shares from the EFI tree species maps[3,4]<br>•New planted forests: The three highest-yielding broadleaved species in the location	•Existing forests baseline & 2050: Based on the shares from the EFI tree species maps[3,4]<br>•New planted forests:<br>The three highest-yielding coniferous species in the location
Management & timber	•No felling for timber production<br>•No thinning is assumed	•Felling for timber production at age of maximum mean annual volume increment[2]<br>•Thinning and wood removal is assumed

Supplementary Table 5 Carbon sequestration modelling - Forest assumptions for broadleaved forests and coniferous plantation forests

Supplementary Table 6 Species distribution model – Species list

Taxonomic group	Species	English common name
Bird	*Botaurus stellaris*	Bittern
Bird	*Caprimulgus europaeus*	Nightjar
Bird	*Locustella naevia*	Grasshopper Warbler
Bird	*Motacilla flava subsp. flavissima*	Yellow Wagtail
Bird	*Phylloscopus sibilatrix*	Wood Warbler
Bird	*Streptopelia turtur*	Turtle Dove
Bird	*Turdus torquatus*	Ring Ouzel
Herptile	*Vipera berus*	Adder
Invertebrate - bee	*Bombus monticola*	Mountain Bumblebee
Invertebrate - beetle	*Cercyon convexiusculus*	Water Beetle sp.
Invertebrate - beetle	*Gnorimus nobilis*	Noble Chafer
Invertebrate - beetle	*Liopterus haemorrhoidalis*	Water Beetle sp.
Invertebrate - butterfly	*Boloria selene*	Small Pearl-Bordered Fritillary
Invertebrate - butterfly	*Coenonympha tullia*	Large Heath
Invertebrate - butterfly	*Cupido minimus*	Small Blue
Invertebrate - butterfly	*Erebia epiphron*	Mountain Ringlet
Invertebrate - butterfly	*Hipparchia semele*	Grayling
Invertebrate - butterfly	*Lasiommata megera*	Wall
Invertebrate - butterfly	*Limenitis camilla*	White Admiral
Invertebrate - butterfly	*Satyrium w-album*	White-Letter Hairstreak
Invertebrate - cricket	*Leptophyes punctatissima*	Speckled Bush Cricket
Invertebrate - cricket	*Metrioptera brachyptera*	Bog Bush Cricket
Invertebrate - moth	*Cossus cossus*	Goat Moth
Invertebrate - moth	*Acronicta psi*	Grey Dagger
Invertebrate - moth	*Allophyes oxyacanthae*	Green-brindled Crescent
Invertebrate - moth	*Dasypolia templi*	Brindled Ochre
Invertebrate - moth	*Xanthorhoe decoloraria*	Red Carpet
Invertebrate - snail	*Cochlodina laminata*	Plaited Door Snail
Invertebrate - snail	*Monacha cantiana*	Kentish Snail
Invertebrate - snail	*Zonitoides excavatus*	Hollowed Glass Snail
Lichen	*Anaptychia ciliaris ciliaris*	Lichen subsp.
Lichen	*Leptogium brebissonii*	Lichen sp.
Lichen	*Parmeliella testacea*	Lichen sp.
Lichen	*Pseudocyphellaria intricata*	Lichen sp.
Lichen	*Usnea articulata*	String-Of-Sausage Lichen
Mammal	*Barbastella barbastellus*	Barbastelle bat
Mammal	*Lepus europaeus*	European hare
Mammal	*Lepus timidus*	Mountain Hare
Mammal	*Martes martes*	Pine Marten
Mammal	*Micromys minutus*	Harvest Mouse
Mammal	*Muscardinus avellanarius*	Hazel Dormouse
Mammal	*Mustela putorius*	Polecat
Mammal	*Myotis bechsteinii*	Bechstein's bat

Mammal	*Nyctalus noctula*	Noctule Bat
Mammal	*Plecotus auritus*	Brown Long-eared Bat
Mammal	*Rhinolophus ferrumequinum*	Greater Horseshoe Bat
Mammal	*Rhinolophus hipposideros*	Lesser Horseshoe Bat
Mammal	*Sciurus vulgaris*	Eurasian red squirrel
Vascular plant	*Anchusa arvensis*	Field bugloss
Vascular plant	*Andromeda polifolia*	Bog Rosemary
Vascular plant	*Arctostaphylos alpinus*	Mountain bearberry
Vascular plant	*Asplenium viride*	Green spleenwort
Vascular plant	*Atriplex laciniata*	Frosted Orache
Vascular plant	*Blysmus rufus*	Saltmarsh Flat-Sedge
Vascular plant	*Cakile maritima*	Sea Rocket
Vascular plant	*Campanula glomerata*	Clustered Bellflower
Vascular plant	*Carex extensa*	Long-Bracted Sedge
Vascular plant	*Carex magellanica*	Tall Bog-Sedge
Vascular plant	*Centaurium pulchellum*	Lesser Centaury
Vascular plant	*Cerastium arvense*	Field Mouse-Ear
Vascular plant	*Cirsium eriophorum*	Woolly Thistle
Vascular plant	*Daphne laureola*	Spurge-Laurel
Vascular plant	*Eriophorum latifolium*	Broad-Leaved Cottongrass
Vascular plant	*Fumaria muralis*	Common Ramping-Fumitory
Vascular plant	*Genista anglica*	Petty Whin
Vascular plant	*Genista tinctoria*	Dyer’s Greenweed
Vascular plant	*Gnaphalium supinum*	Dwarf Cudweed
Vascular plant	*Goodyera repens*	Creeping Lady’s-Tresses
Vascular plant	*Hypericum elodes*	Marsh St John’s-Wort
Vascular plant	*Lamium hybridum*	Cut-Leaved Dead-Nettle
Vascular plant	*Leymus arenarius*	Lyme Grass
Vascular plant	*Lycopodium clavatum*	Stag’s-Horn Clubmoss
Vascular plant	*Neottia nidus-avis*	Bird’s-Nest Orchid
Vascular plant	*Ornithopus perpusillus*	Bird’s-Foot
Vascular plant	*Orthilia secunda*	Serrated Wintergreen
Vascular plant	*Oxyria digyna*	Mountain Sorrel
Vascular plant	*Pyrola media*	Intermediate Wintergreen
Vascular plant	*Radiola linoides*	Allseed
Vascular plant	*Ranunculus omiophyllus*	Round-Leaved Crowfoot
Vascular plant	*Saxifraga tridactylites*	Rue-Leaved Saxifrage
Vascular plant	*Silaum silaus*	Pepper-Saxifrage
Vascular plant	*Thymus pulegioides*	Large Thyme
Vascular plant	*Tilia cordata*	Small-Leaved Lime
Vascular plant	*Trifolium fragiferum*	Strawberry Clover
Vascular plant	*Trifolium striatum*	Knotted Clover
Vascular plant	*Vaccinium microcarpum*	Small Cranberry

Supplementary Table 6 Species included in the species distribution model part of the biodiversity measure

Supplementary Table 7 NBN atlas data partners

Data partners for Botaurus stellaris:

- Bristol Regional Environmental Records Centre (2023). BRERC Notable Species records within the last 10 years. Occurrence dataset on the NBN Atlas. https://doi.org/10.15468/vntgox
- British Trust for Ornithology (2023). BTO First Atlas of Breeding Birds in Britain and Ireland: 1968-1972. Occurrence dataset on the NBN Atlas. https://doi.org/10.15468/cfigc5
- Leicestershire and Rutland Environmental Records Centre (2023). Leicestershire and Rutland Environmental Records Centre records 2000-2009. Occurrence dataset on the NBN Atlas. https://doi.org/10.15468/cs2zzf
- Leicestershire and Rutland Environmental Records Centre (2023). Leicestershire and Rutland Environmental Records Centre records 2010-2014. Occurrence dataset on the NBN Atlas. https://doi.org/10.15468/9n92x3
- Leicestershire and Rutland Environmental Records Centre (2023). Leicestershire and Rutland Environmental Records Centre records pre 2000. Occurrence dataset on the NBN Atlas. https://doi.org/10.15468/res3cx
- Leicestershire and Rutland Environmental Records Centre (2023). Leicestershire and Rutland Environmental Records Centre records 2015-2019. Occurrence dataset on the NBN Atlas. https://doi.org/10.15468/dues93
- Records provided by Manx National Heritage, accessed through NBN Atlas website.
- SEWBReC (2023). NRW Regional Data: South East Wales Sensitive species. Occurrence dataset on the NBN Atlas. https://doi.org/10.15468/zk26dm
- Records provided by Fife Nature Records Centre, accessed through NBN Atlas website.
- The Scottish Ornithologists' Club (2023). North East Scotland Breeding Bird Atlas 2002-06. Occurrence dataset on the NBN Atlas. https://doi.org/10.15468/hny7nf
- Cambridgeshire & Peterborough Environmental Records Centre (2023). CPERC Combined Dataset. Occurrence dataset on the NBN Atlas. https://doi.org/10.15468/npthhv
- Suffolk Biodiversity Information Service (2023). Suffolk Biodiversity Information Service (SBIS) Dataset. Occurrence dataset on the NBN Atlas. https://doi.org/10.15468/ab4vwo
- Records provided by Royal Society for the Protection of Birds, accessed through NBN Atlas website.
- Records provided by John Muir Trust, accessed through NBN Atlas website.
- Cofnod (2023). Miscellaneous records held on the Cofnod database. Occurrence dataset on the NBN Atlas. https://doi.org/10.15468/hcgqsi
- BIS for Powys and Brecon Beacons National Park (2023). Mongomeryshire Wildlife Trust records held by BIS. Occurrence dataset on the NBN Atlas. https://doi.org/10.15468/vozyfp
- Records provided by NatureSpot, accessed through NBN Atlas website.
- Scottish Ornithologists' Club, The (2023). Highland bird data 2002 - 2003 - non-breeding records. Occurrence dataset on the NBN Atlas. https://doi.org/10.15468/ovr9nu
- BIS for Powys and Brecon Beacons National Park (2023). Radnorshire Wildlife Trust records held by BIS. Occurrence dataset on the NBN Atlas. https://doi.org/10.15468/hxqokw
- Records provided by Leicestershire and Rutland Environmental Records Centre, accessed through NBN Atlas website.
- BIS for Powys and Brecon Beacons National Park (2023). Miscellaneous records held by BIS. Occurrence dataset on the NBN Atlas. https://doi.org/10.15468/mo7peo
- Merseyside BioBank (2020) www.merseysidebiobank.org.uk. https://doi.org/10.15468/ar0p6s
- Murray, R.D., Andrews, I.J. & Holling, M. 2019. Birds in South-east Scotland 2007-13: a tetrad atlas of the birds of Lothian and Borders. The Scottish Ornithologists' Club, Aberlady. https://doi.org/10.15468/28drbf
- Manx National Heritage Library and Archives (2023). Calf of Man (Isle of Man) Bird Observatory Daily Logs, 1959-2004. Occurrence dataset on the NBN Atlas. https://doi.org/10.15468/rzpbo5
- Records provided by Greater Manchester Ecology Unit, accessed through NBN Atlas website.
- Records provided by All taxa records for Leicestershire and Rutland, accessed through NBN Atlas website. https://doi.org/10.15468/i46are
- BIS for Powys & Brecon Beacons National Park (2023). Natural Resources Wales Regional Data: Mid-Wales. Occurrence dataset on the NBN Atlas. https://doi.org/10.15468/whj6d7
- British Trust for Ornithology (2023). BTO Second Atlas of Breeding Birds in Britain and Ireland: 1988-1991. Occurrence dataset on the NBN Atlas. https://doi.org/10.15468/cisnhd
- Records provided by Cambridgeshire & Peterborough Environmental Records Centre, accessed through NBN Atlas website.
- Cumbria Biodiversity Data Centre (2023). Tullie House Museum Natural History Collections. Occurrence dataset on the NBN Atlas. https://doi.org/10.15468/epewfs
- Natural Resources Wales. (2023) Stackpole National Nature Reserve Species Inventory and Ad-hoc Sightings from Across Pembrokeshire. Occurrence dataset accessed through the NBNAtlas. https://doi.org/10.15468/k6hvb8
- Records provided by Cumbria Biodiversity Data Centre, accessed through NBN Atlas website.
- Records provided by West Wales Biodiversity Information Centre, accessed through NBN Atlas website.
- Records provided by BTO, accessed through NBN Atlas website
- Records provided by Bristol Regional Environmental Records Centre, accessed through NBN Atlas website.
- Records provided by Lancashire Environment Record Network, accessed through NBN Atlas website.
- Records provided by Dorset Environmental Records Centre, accessed through NBN Atlas website.
- Records provided by BTO, accessed through NBN Atlas website
- West Wales Biodiversity Information Centre (2023). NRW Regional Data: all taxa (excluding sensitive species), West Wales. Occurrence dataset on the NBN Atlas. https://doi.org/10.15468/q3d1hl
- Lancashire Environment Record Network (2023). LERN Records. Occurrence dataset on the NBN Atlas. https://doi.org/10.15468/esxc9a
- Records provided by Rotherham Biological Records Centre, accessed through NBN Atlas website.
- Royal Society for the Protection of Birds (2023). RSPB Annual Reserve Avian Monitoring. Occurrence dataset on the NBN Atlas. https://doi.org/10.15468/ssn1l9
- World Museum, National Museums Liverpool (2020). British bird study skins in the collections of World Museum, National Museums Liverpool. https://doi.org/10.15468/tekr68

- West Wales Biodiversity Information Centre (2023). West Wales Online Records. Occurrence dataset on the NBN Atlas
- RECORD (2023). RECORD Aves Data. Occurrence dataset on the NBN Atlas. https://doi.org/10.15468/pudf3y
- Staffordshire Ecological Record (2023). Data from Defra Family Organisations supplied to Staffordshire Ecological Record. Occurrence dataset on the NBN Atlas. https://doi.org/10.15468/giebpp
- Norfolk Biodiversity Information Service (2023). NBIS Records to December 2016. Occurrence dataset on the NBN Atlas. https://doi.org/10.15468/jca5lo
- Records provided by BTO, accessed through NBN Atlas website
- Records provided by BIS for Powys & Brecon Beacons National Park, accessed through NBN Atlas website.
- Records provided by BTO, accessed through NBN Atlas website
- Records provided by BTO, accessed through NBN Atlas website
- Environmental Records Information Centre North East (2023). ERIC NE Combined dataset to 2017. Occurrence dataset on the NBN Atlas
- Records provided by Suffolk Biodiversity Information Service, accessed through NBN Atlas website.
- Records provided by World Museum, National Museums Liverpool, accessed through NBN Atlas website.
- Gloucestershire Centre for Environmental Records (2023). Gloucestershire Historic Wildlife Sightings prior to 1st Jan 2000. Occurrence dataset on the NBN Atlas. https://doi.org/10.15468/dgf5es
- Records provided by Natural Resources Wales, accessed through NBN Atlas website.
- Records provided by North East Scotland Biological Records Centre, accessed through NBN Atlas website.
- Rotherham Biological Records Centre (2023). Non-sensitive Records from all taxonomic groups. Occurrence dataset on the NBN Atlas. https://doi.org/10.15468/d3tufo
- Shropshire Ecological Data Network (2023). Shropshire Ecological Data Network database. Occurrence dataset on the NBN Atlas. https://doi.org/10.15468/5v5pvk
- Records provided by Shropshire Ecological Data Network, accessed through NBN Atlas website.
- Dorset Environmental Records Centre (2023). Dorset SSSI Species Records 1952 - 2004 (Natural England). Occurrence dataset on the NBN Atlas. https://doi.org/10.15468/vcjzts
- Greater Manchester Ecology Unit (2023). Bird Records held by Greater Manchester Ecology Unit. Occurrence dataset on the NBN Atlas
- Records provided by South East Wales Biodiversity Records Centre, accessed through NBN Atlas website.
- Bristol Regional Environmental Records Centre (2023). BEREC species records from all years at full resolution excluding Notable Species within the last 10 years. Occurrence dataset on the NBN Atlas. https://doi.org/10.15468/h1ln5p
- Natural History Museum (2023). Data Portal query on 1 resources https://doi.org/10.5519/qd.i4tk4lrk. https://doi.org/https://doi.org/10.5519/0002965
- Records provided by Environmental Records Information Centre North East, accessed through NBN Atlas website.
- Records provided by Merseyside BioBank, accessed through NBN Atlas website.
- Records provided by Isle of Wight Local Records Centre, accessed through NBN Atlas website.
- Records provided by Ministry of Justice, accessed through NBN Atlas website.
- Records provided by Norfolk Biodiversity Information Service, accessed through NBN Atlas website.
- Isle of Wight Local Records Centre (2023). Isle of Wight Notable Species. Occurrence dataset on the NBN Atlas. https://doi.org/10.15468/sm4ety
- Records provided by SEWBReC Birds (South East Wales), accessed through NBN Atlas website. https://doi.org/10.15468/qfainq
- John Muir Trust (2023). Species Records for John Muir Trust Properties Nevis, Sandwood, Quinag and Schiehallion 2010. Occurrence dataset on the NBN Atlas. https://doi.org/10.15468/n9dwn0
- Records provided by National Trust, accessed through NBN Atlas website.
- Records provided by North East Scotland Birds (1883-2010), accessed through NBN Atlas website. https://doi.org/10.15468/o1okcz
- Staffordshire Ecological Record (2023). SER Species-based Surveys. Occurrence dataset on the NBN Atlas. https://doi.org/10.15468/q8qen3
- Sheffield Bird Study Group (2023). Sheffield Bird Study Group (Yorkshire records). Occurrence dataset on the NBN Atlas. https://doi.org/10.15468/ytwnad
- National Trust (2023). National Trust Species Records. Occurrence dataset on the NBN Atlas. https://doi.org/10.15468/opc6g1
- Records provided by Scottish Ornithologists' Club, The, accessed through NBN Atlas website.
- British Trust for Ornithology (2023). BTO First Atlas of Wintering Birds in Britain and Ireland: 1981/82-1983/84. Occurrence dataset on the NBN Atlas. https://doi.org/10.15468/huodzs
- Records provided by Natural History Museum, London, accessed through NBN Atlas website.
- RSPB (2023). Bittern Annual Surveys in Britain 1990-2019. Occurrence dataset on the NBN Atlas. https://doi.org/10.15468/6vvmv4
- Fife Nature Records Centre (2023). Fife Nature Records Centre combined dataset. Occurrence dataset on the NBN Atlas. https://doi.org/10.15468/ccc1ip
- SEWBReC (2023). Dr Mary Gillham Archive Project. Occurrence dataset on the NBN Atlas. https://doi.org/10.15468/ajv47f
- Ministry of Justice (2023). Species found in the NOMS estate 2005 - Present. Occurrence dataset on the NBN Atlas. https://doi.org/10.15468/wcx4is
- Records provided by Sheffield Bird Study Group, accessed through NBN Atlas website.
- Merseyside BioBank (2020) www.merseysidebiobank.org.uk. https://doi.org/10.15468/iou2ld
- West Wales Biodiversity Information Centre (2023). WTSWW Data: All Taxa (West Wales). Occurrence dataset on the NBN Atlas. https://doi.org/10.15468/gaakk2
- Records provided by Yorkshire Wildlife Trust - Non-sensitive records from all taxonomic groups, accessed through NBN Atlas website. https://doi.org/10.15468/2razk5
- Records provided by Record, accessed through NBN Atlas website.
- Cofnod – North Wales Environmental Information Service (2023). NRW Regional Data: North Wales. Occurrence dataset on the NBN Atlas. https://doi.org/10.15468/krljpu
- Records provided by Staffordshire Ecological Record, accessed through NBN Atlas website.
- Greater Manchester Ecology Unit (2023). Distribution of Species of Conservation Interest in Greater Manchester. Occurrence dataset on the NBN Atlas
- Records provided by Yorkshire Wildlife Trust, accessed through NBN Atlas website.
- World Museum, National Museums Liverpool (2023). British records in the osteology collections of World Museum, National Museums Liverpool. Occurrence dataset on the NBN Atlas
- Records provided by Cofnod – North Wales Environmental Information Service, accessed through NBN Atlas website.
- Records provided by British Trust for Ornithology, accessed through NBN Atlas website.
- Records provided by Gloucestershire Centre for Environmental Records, accessed through NBN Atlas website.

Data partners for Caprimulgus europaeus:

- Bristol Regional Environmental Records Centre (2023). BRERC Notable Species records within the last 10 years. Occurrence dataset on the NBN Atlas. https://doi.org/10.15468/vntgox
- Records provided by Bat Conservation Trust, accessed through NBN Atlas website.
- Records provided by Royal Horticultural Society, accessed through NBN Atlas website.
- Natural Resources Wales. (2023) Ty Canol National Nature Reserve (NNR) Species Inventory. Occurrence dataset accessed through the NBNAtlas. https://doi.org/10.15468/shxquu
- British Trust for Ornithology (2023). BTO First Atlas of Breeding Birds in Britain and Ireland: 1968-1972. Occurrence dataset on the NBN Atlas. https://doi.org/10.15468/cfigc5
- Leicestershire and Rutland Environmental Records Centre (2023). Leicestershire and Rutland Environmental Records Centre records 2000-2009. Occurrence dataset on the NBN Atlas. https://doi.org/10.15468/cs2zzf
- Leicestershire and Rutland Environmental Records Centre (2023). Leicestershire and Rutland Environmental Records Centre records 2010-2014. Occurrence dataset on the NBN Atlas. https://doi.org/10.15468/9n92x3
- Leicestershire and Rutland Environmental Records Centre (2023). Leicestershire and Rutland Environmental Records Centre records pre 2000. Occurrence dataset on the NBN Atlas. https://doi.org/10.15468/res3cx
- Malcolm Storey (2023). http://www.bioimages.org.uk/ Malcolm Storey personal records and images. Occurrence dataset on the NBN Atlas
- Records provided by Argyll Bird Club, accessed through NBN Atlas website.
- Leicestershire and Rutland Environmental Records Centre (2023). Leicestershire and Rutland Environmental Records Centre records 2015-2019. Occurrence dataset on the NBN Atlas. https://doi.org/10.15468/dues93
- Records provided by Manx National Heritage, accessed through NBN Atlas website.
-Reproduced by permission of the Bird Conservation Targeting Project. © Bird Conservation Targeting Project, 2010 (a partnership between the British Trust for Ornithology (BTO), the Centre for Environmental Data and Recording (CEDaR), the Countryside Council for Wales (CCW), the Department of Agriculture and Rural Development (DARD), Forestry Commission England (FCE), Forestry Commission Wales (FCW), Forest Service (FS), Natural England (NE), Northern Ireland Environment Agency, the RSPB and Scottish Natural Heritage (SNH)). All rights reserved. The Bird Conservation Targeting Project partners are grateful to the contributions of the data providers listed at www.rspb.org.uk/targeting, and to the many volunteers who collected these data. https://doi.org/10.15468/y4z6zz
- The Scottish Ornithologists' Club (2023). North East Scotland Breeding Bird Atlas 2002-06. Occurrence dataset on the NBN Atlas. https://doi.org/10.15468/hny7nf
- SEWBReC (2023). NRW Regional Data: South East Wales Non-sensitive species. Occurrence dataset on the NBN Atlas. https://doi.org/10.15468/g7xxs8
- Suffolk Biodiversity Information Service (2023). Suffolk Biodiversity Information Service (SBIS) Dataset. Occurrence dataset on the NBN Atlas. https://doi.org/10.15468/ab4vwo
- Cambridgeshire & Peterborough Environmental Records Centre (2023). CPERC Combined Dataset. Occurrence dataset on the NBN Atlas. https://doi.org/10.15468/npthhv
- Records provided by The Wildlife Information Centre, accessed through NBN Atlas website.
- Records provided by Royal Society for the Protection of Birds, accessed through NBN Atlas website.
- Records provided by John Muir Trust, accessed through NBN Atlas website.
- Cofnod (2023). Miscellaneous records held on the Cofnod database. Occurrence dataset on the NBN Atlas. https://doi.org/10.15468/hcgqsi
- BIS for Powys and Brecon Beacons National Park (2023). Mongomeryshire Wildlife Trust records held by BIS. Occurrence dataset on the NBN Atlas. https://doi.org/10.15468/vozyfp
- Murray, R.D., Andrews, I.J. & Holling, M. 2019. Birds in South-east Scotland 2007-13: a tetrad atlas of the birds of Lothian and Borders. The Scottish Ornithologists' Club, Aberlady. https://doi.org/10.15468/9xhy5h
- BIS for Powys and Brecon Beacons National Park (2023). Miscellaneous records held by BIS. Occurrence dataset on the NBN Atlas. https://doi.org/10.15468/mo7peo
- Records provided by Leicestershire and Rutland Environmental Records Centre, accessed through NBN Atlas website.
- Merseyside BioBank (2020) www.merseysidebiobank.org.uk. https://doi.org/10.15468/ar0p6s
- BIS for Powys and Brecon Beacons National Park (2023). Brecknock Wildlife Trust (Now WTSWW Brecknockhire) records held by BIS. Occurrence dataset on the NBN Atlas. https://doi.org/10.15468/hd7pvq
- Scottish Ornithologists' Club, The (2023). Highland Breeding Bird Data 2002 - 2003 - sensitive breeding species. Occurrence dataset on the NBN Atlas. https://doi.org/10.15468/iddlbd
- British Trust for Ornithology (2023). Nightjar national breeding surveys in Britain. Occurrence dataset on the NBN Atlas. https://doi.org/10.15468/yjrm3w
- Manx National Heritage Library and Archives (2023). Calf of Man (Isle of Man) Bird Observatory Daily Logs, 1959-2004. Occurrence dataset on the NBN Atlas. https://doi.org/10.15468/rzpbo5
- Records provided by Greater Manchester Ecology Unit, accessed through NBN Atlas website.
- BIS for Powys & Brecon Beacons National Park (2023). Natural Resources Wales Regional Data: Mid-Wales. Occurrence dataset on the NBN Atlas. https://doi.org/10.15468/whj6d7
- British Trust for Ornithology (2023). BTO Second Atlas of Breeding Birds in Britain and Ireland: 1988-1991. Occurrence dataset on the NBN Atlas. https://doi.org/10.15468/cisnhd
- The British Association for Shooting and Conservation (2023). UK casual records from members of BASC - 1980 onwards. Occurrence dataset on the NBN Atlas
- National Bat Monitoring Programme [date accessed] Sunset/Sunrise Survey, Bat Conservation Trust. https://doi.org/10.15468/brmbw2
- Cumbria Biodiversity Data Centre (2023). Tullie House Museum Natural History Collections. Occurrence dataset on the NBN Atlas. https://doi.org/10.15468/epewfs
- Staffordshire Ecological Record (2023). SER Site-based Surveys. Occurrence dataset on the NBN Atlas. https://doi.org/10.15468/h2yko0
- Records provided by Cambridgeshire & Peterborough Environmental Records Centre, accessed through NBN Atlas website.
- Records provided by Cumbria Biodiversity Data Centre, accessed through NBN Atlas website.
- Natural Resources Wales. (2023) Stackpole National Nature Reserve Species Inventory and Ad-hoc Sightings from Across Pembrokeshire. Occurrence dataset accessed through the NBNAtlas. https://doi.org/10.15468/k6hvb8
- Records provided by West Wales Biodiversity Information Centre, accessed through NBN Atlas website.

- Records provided by Bristol Regional Environmental Records Centre, accessed through NBN Atlas website.
- Records provided by BTO, accessed through NBN Atlas website
- Royal Society for the Protection of Birds (2023). Nightjar breeding season surveys in Dumfries and Galloway between 1981 and 2011. Occurrence dataset on the NBN Atlas. https://doi.org/10.15468/d3oo4b
- Records provided by Lancashire Environment Record Network, accessed through NBN Atlas website.
- Records provided by Dorset Environmental Records Centre, accessed through NBN Atlas website.
- Records provided by BTO, accessed through NBN Atlas website
- Staffordshire Ecological Record (2023). Staffordshire Wildlife Trust Nature Reserves Inventory. Occurrence dataset on the NBN Atlas. https://doi.org/10.15468/vhdows
- Data supplied by Sheffield and Rotherham Wildlife Trust. https://doi.org/10.15468/x3wdpp
- West Wales Biodiversity Information Centre (2023). NRW Regional Data: all taxa (excluding sensitive species), West Wales. Occurrence dataset on the NBN Atlas. https://doi.org/10.15468/q3d1hl
- Lancashire Environment Record Network (2023). LERN Records. Occurrence dataset on the NBN Atlas. https://doi.org/10.15468/esxc9a
- The Wildlife Information Centre (2023). City of Edinburgh Natural Heritage Service - General Public Records. Occurrence dataset on the NBN Atlas. https://doi.org/10.15468/mfkmtz
- Records provided by Rotherham Biological Records Centre, accessed through NBN Atlas website.
- Records provided by The British Association for Shooting and Conservation, accessed through NBN Atlas website.
- Natural England iRecord Surveys (2023). https://doi.org/10.15468/i7x5ca
- Argyll Bird Club (2023). Bird records for Argyll for the years 2002 and 2003. Occurrence dataset on the NBN Atlas. https://doi.org/10.15468/uilybi
- Royal Society for the Protection of Birds (2023). RSPB Annual Reserve Avian Monitoring. Occurrence dataset on the NBN Atlas. https://doi.org/10.15468/ssn1l9
- World Museum, National Museums Liverpool (2020). British bird study skins in the collections of World Museum, National Museums Liverpool. https://doi.org/10.15468/tekr68
- West Wales Biodiversity Information Centre (2023). West Wales Online Records. Occurrence dataset on the NBN Atlas
- RECORD (2023). RECORD Aves Data. Occurrence dataset on the NBN Atlas. https://doi.org/10.15468/pudf3y
- Staffordshire Ecological Record (2023). Data from Defra Family Organisations supplied to Staffordshire Ecological Record. Occurrence dataset on the NBN Atlas. https://doi.org/10.15468/giebpp
- Records provided by Malcolm Storey, accessed through NBN Atlas website.
- Norfolk Biodiversity Information Service (2023). NBIS Records to December 2016. Occurrence dataset on the NBN Atlas. https://doi.org/10.15468/jca5lo
- Records provided by BTO, accessed through NBN Atlas website
- Records provided by BIS for Powys & Brecon Beacons National Park, accessed through NBN Atlas website.
- Records provided by BTO, accessed through NBN Atlas website
- Records provided by BTO, accessed through NBN Atlas website
- Environmental Records Information Centre North East (2023). ERIC NE Combined dataset to 2017. Occurrence dataset on the NBN Atlas
- Records provided by World Museum, National Museums Liverpool, accessed through NBN Atlas website.
- Records provided by Suffolk Biodiversity Information Service, accessed through NBN Atlas website.
- Gloucestershire Centre for Environmental Records (2023). Gloucestershire Historic Wildlife Sightings prior to 1st Jan 2000. Occurrence dataset on the NBN Atlas. https://doi.org/10.15468/dgf5es
- Records provided by Natural Resources Wales, accessed through NBN Atlas website.
- Records provided by North East Scotland Biological Records Centre, accessed through NBN Atlas website.
- Natural Resources Wales. (2023) Welsh Invertebrate Database (WID). Occurrence dataset accessed through the NBNAtlas. https://doi.org/10.15468/bv8fcj
- Rotherham Biological Records Centre (2023). Non-sensitive Records from all taxonomic groups. Occurrence dataset on the NBN Atlas. https://doi.org/10.15468/d3tufo
- Shropshire Ecological Data Network (2023). Shropshire Ecological Data Network database. Occurrence dataset on the NBN Atlas. https://doi.org/10.15468/5v5pvk
- Records provided by Shropshire Ecological Data Network, accessed through NBN Atlas website.
- Dorset Environmental Records Centre (2023). Dorset SSSI Species Records 1952 - 2004 (Natural England). Occurrence dataset on the NBN Atlas. https://doi.org/10.15468/vcjzts
- Greater Manchester Ecology Unit (2023). Bird Records held by Greater Manchester Ecology Unit. Occurrence dataset on the NBN Atlas
- Records provided by South East Wales Biodiversity Records Centre, accessed through NBN Atlas website.
- Royal Horticultural Society (2023). RHS monitoring of native and naturalised plants and animals at its gardens and surrounding areas. Occurrence dataset on the NBN Atlas. https://doi.org/10.15468/mjksef
- Bristol Regional Environmental Records Centre (2023). BEREC species records from all years at full resolution excluding Notable Species within the last 10 years. Occurrence dataset on the NBN Atlas. https://doi.org/10.15468/h1ln5p
- Records provided by Natural England, accessed through NBN Atlas website.
- BIS for Powys & Brecon Beacons National Park (2023). Brecknock county birds records. Occurrence dataset on the NBN Atlas. https://doi.org/10.15468/2rd8nh
- Natural History Museum (2023). Data Portal query on 1 resource https://doi.org/10.5519/qd.i4tk4lrk. https://doi.org/https://doi.org/10.5519/0002965
- Records provided by Merseyside BioBank, accessed through NBN Atlas website.
- Records provided by Environmental Records Information Centre North East, accessed through NBN Atlas website.
- Records provided by Isle of Wight Local Records Centre, accessed through NBN Atlas website.
- Records provided by Sheffield and Rotherham Wildlife Trust, accessed through NBN Atlas website.
- Isle of Wight Local Records Centre (2023). Isle of Wight Notable Species. Occurrence dataset on the NBN Atlas. https://doi.org/10.15468/sm4ety
- Records provided by Norfolk Biodiversity Information Service, accessed through NBN Atlas website.
- Records provided by SEWBReC Birds (South East Wales), accessed through NBN Atlas website. https://doi.org/10.15468/qfainq
- John Muir Trust (2023). Species Records for John Muir Trust Properties Nevis, Sandwood, Quinag and Schiehallion 2010. Occurrence dataset on the NBN Atlas. https://doi.org/10.15468/n9dwn0
- Records provided by National Trust, accessed through NBN Atlas website.
- Records provided by North East Scotland Birds (1883-2010), accessed through NBN Atlas website. https://doi.org/10.15468/o1okcz

- Staffordshire Ecological Record (2023). SER Species-based Surveys. Occurrence dataset on the NBN Atlas. https://doi.org/10.15468/q8qen3
- Sheffield Bird Study Group (2023). Sheffield Bird Study Group (Yorkshire records). Occurrence dataset on the NBN Atlas. https://doi.org/10.15468/ytwnad
- National Trust (2023). National Trust Species Records. Occurrence dataset on the NBN Atlas. https://doi.org/10.15468/opc6g1
- Records provided by Scottish Ornithologists' Club, The, accessed through NBN Atlas website.
- Records provided by Natural History Museum, London, accessed through NBN Atlas website.
- SEWBReC (2023). Dr Mary Gillham Archive Project. Occurrence dataset on the NBN Atlas. https://doi.org/10.15468/ajv47f
- World Museum, National Museums Liverpool (2023). British bird eggs in the collections of World Museum, National Museums Liverpool. https://doi.org/10.15468/n75arp
- Records provided by Sheffield Bird Study Group, accessed through NBN Atlas website.
- Dorset Environmental Records Centre (2023). Dorset Sites of Nature Conservation Interest (SNCI) species records pre 2000. Occurrence dataset on the NBN Atlas. https://doi.org/10.15468/qyg29v
- Reproduced by permission of the Bird Conservation Targeting Project. © Bird Conservation Targeting Project, 2010 (a partnership between the British Trust for Ornithology (BTO), the Centre for Environmental Data and Recording (CEDaR), the Countryside Council for Wales (CCW), the Department of Agriculture and Rural Development (DARD), Forestry Commission England (FCE), Forestry Commission Wales (FCW), Forest Service (FS), Natural England (NE), Northern Ireland Environment Agency, the RSPB and Scottish Natural Heritage (SNH)). All rights reserved.; The Bird Conservation Targeting Project partners are grateful to the contributions of the data providers listed at www.rspb.org.uk/targeting", and to the many volunteers who collected these data. https://doi.org/10.15468/mep71d
- West Wales Biodiversity Information Centre (2023). WTSWW Data: All Taxa (West Wales). Occurrence dataset on the NBN Atlas. https://doi.org/10.15468/gaakk2
- Records provided by Yorkshire Wildlife Trust - Non-sensitive records from all taxonomic groups, accessed through NBN Atlas website. https://doi.org/10.15468/2razk5
- Records provided by Record, accessed through NBN Atlas website.
- Cofnod – North Wales Environmental Information Service (2023). NRW Regional Data: North Wales. Occurrence dataset on the NBN Atlas. https://doi.org/10.15468/krljpu
- Records provided by Staffordshire Ecological Record, accessed through NBN Atlas website.
- Greater Manchester Ecology Unit (2023). Distribution of Species of Conservation Interest in Greater Manchester. Occurrence dataset on the NBN Atlas
- Records provided by Yorkshire Wildlife Trust, accessed through NBN Atlas website.
- Records provided by Cofnod – North Wales Environmental Information Service, accessed through NBN Atlas website.
- World Museum, National Museums Liverpool (2023). British records in the osteology collections of World Museum, National Museums Liverpool. Occurrence dataset on the NBN Atlas
- Records provided by British Trust for Ornithology, accessed through NBN Atlas website.
- Records provided by Gloucestershire Centre for Environmental Records, accessed through NBN Atlas website.
Due to the length of the table that contains the data partners for all species, the remaining species can be found in the full table via https://figshare.com/articles/dataset/CarbonFoodNature_TradeOffs_-_Source_data/29618120

Supplementary Table 7 NBN atlas data partners that collected the species occurrence data used in the species distribution model. This version of the table includes the data partners for two species, the remaining species and data partners can be found in the full length table on https://figshare.com/articles/dataset/CarbonFoodNature_TradeOffs_-_Source_data/29618120

Supplementary References

1. Forest Research. Forestry Statistics 2023, Chapter 1: Woodland Area & Planting. in *forest* (2023).
2. Bradley, R. T., Christie, J. M. & Johnston, D. R. *Forest Management Tables - Forestry Commission Booklet No. 16*. (Her Majesty's Stationery Office, 1966).
3. Brus, D. J. *et al.* Statistical mapping of tree species over Europe. *Eur. J. For. Res.* **131**, 145–157 (2011).
4. Nabuurs, G. J. *et al.* European Forest Institute - Tree species maps for European forests. https://efi.int/knowledge/maps/treespecies.
