# Supplementary material for: Strategic land reallocation enhances carbon sequestration and biodiversity protection without compromising agricultural productivity in Great Britain
Source: Commun Earth Environ. 2025 Sep 29;6(1):770. doi: 10.1038/s43247-025-02728-w (PMC12479351; doi:10.1038/s43247-025-02728-w)
Supplement: Supplementary file 3 — Reporting summary [file 43247_2025_2728_MOESM3_ESM.pdf]

nature portfolio

Corresponding author(s): Sarah S. Gall

Last updated by author(s): 24/07/2025

Reporting Summary

Nature Portfolio wishes to improve the reproducibility of the work that we publish. This form provides structure for consistency and transparency in reporting. For further information on Nature Portfolio policies, see our Editorial Policies and the Editorial Policy Checklist.

Statistics

For all statistical analyses, confirm that the following items are present in the figure legend, table legend, main text, or Methods section.

n/a	Confirmed	
☒	☐	The exact sample size (*n*) for each experimental group/condition, given as a discrete number and unit of measurement
☒	☐	A statement on whether measurements were taken from distinct samples or whether the same sample was measured repeatedly
☒	☐	The statistical test(s) used AND whether they are one- or two-sided<br>*Only common tests should be described solely by name; describe more complex techniques in the Methods section.*
☒	☐	A description of all covariates tested
☒	☐	A description of any assumptions or corrections, such as tests of normality and adjustment for multiple comparisons
☒	☐	A full description of the statistical parameters including central tendency (e.g. means) or other basic estimates (e.g. regression coefficient) AND variation (e.g. standard deviation) or associated estimates of uncertainty (e.g. confidence intervals)
☒	☐	For null hypothesis testing, the test statistic (e.g. *F*, *t*, *r*) with confidence intervals, effect sizes, degrees of freedom and *P* value noted<br>*Give P values as exact values whenever suitable.*
☒	☐	For Bayesian analysis, information on the choice of priors and Markov chain Monte Carlo settings
☒	☐	For hierarchical and complex designs, identification of the appropriate level for tests and full reporting of outcomes
☒	☐	Estimates of effect sizes (e.g. Cohen's *d*, Pearson's *r*), indicating how they were calculated

Our web collection on statistics for biologists contains articles on many of the points above.

Software and code

Policy information about availability of computer code

Data collection	In some instances code was use to facilitate the download of big datasets.
Data analysis	The code used for creating the model, preparing the input data and analysing the model results is available on GitHub as stated in the Code availability Statement on the manuscript

For manuscripts utilizing custom algorithms or software that are central to the research but not yet described in published literature, software must be made available to editors and reviewers. We strongly encourage code deposition in a community repository (e.g. GitHub). See the Nature Portfolio guidelines for submitting code & software for further information.

Data

Policy information about availability of data

All manuscripts must include a data availability statement. This statement should provide the following information, where applicable:

- Accession codes, unique identifiers, or web links for publicly available datasets
- A description of any restrictions on data availability
- For clinical datasets or third party data, please ensure that the statement adheres to our policy

All datasets used in this study are cited in the relevant sections. The land cover raster can be downloaded from the UK Centre for Ecology & Hydrology via the EDINA Environment Digimap service (https://digimap.edina.ac.uk/environment) or https://www.ceh.ac.uk/data/ukceh-land-cover-maps. Spatial data for drained peatlands and peaty soils were downloaded for England from Natural England via https://www.data.gov.uk/dataset/9d494f48-f0d7-4333-96f0-8b736ac8fb18/peaty-soils-

location1 and https://www.data.gov.uk/dataset/b12f420a-d9f1-4966-aa3e-0f6e680e3875/moorland-deep-peat-ap-status1, for Wales from UKCEH via https://catalogue.ceh.ac.uk/documents/58139ce6-63f9-4444-9f77-fc7b5dcc00d8 and for Scotland from Scottish Natural Heritage via https://opendata.nature.scot/datasets/snh::carbon-and-peatland-2016-map/explore. Tree species maps for European forests were downloaded from the European Forest Institute via https://efi.int/knowledge/maps/treespecies. Tree species and location-specific yield class potential data can be obtained from the Forest Research Ecological site classification tool via http://www.forestdss.org.uk/geoforestdss/esc4.jsp. Yield class specific carbon sequestration data was obtained from the Woodland carbon code Lookup tables in the Carbon calculation spreadsheet, which can be downloaded via https://www.woodlandcarboncode.org.uk/landowners-apply/template-documents. Forestry production and trade data was downloaded from FAOSTAT and found here: https://www.fao.org/faostat/en/#data/FO. The MapSPAM raster data on crop production areas was downloaded via https://mapspam.info/. Location-specific attainable arable yields can be downloaded from FAO GAEZv4 via https://gaez.fao.org/pages/data-viewer. Statistics on livestock patterns can be downloaded from FAOSTAT via https://www.fao.org/faostat/en/#data/EK. Producer prices for agricultural products can be downloaded from FAOSTAT via https://www.fao.org/faostat/en/#data/PP. Total harvested area and average livestock yields can be downloaded from the Crops and livestock products dataset from FAOSTAT via https://www.fao.org/faostat/en/#data/QCL. The Suitability of global land area for pasture (FGGD) raster data can be downloaded from FAO via https://data.apps.fao.org/catalog/iso/2b357400-891a-11db-b9b2-000d939bc5d8. Data on carbon densities for different tree species can be downloaded from the Global wood densities database via https://datadryad.org/stash/dataset/doi:10.5061/dryad.234. The species occurrence data was downloaded from the NBN atlas database via https://nbnatlas.org/. Rainfall and temperature data from the HadUK-Grid Gridded Climate Observations data can be downloaded from the Centre for Environmental Data Analysis (CEDA) Archive via https://catalogue.ceda.ac.uk/uuid/bbca3267dc7d4219af484976734c9527/. Data on the proportion of native species for the habitat condition calculations can be downloaded from the PREDICTS database from the data portal of the Natural History Museum via https://data.nhm.ac.uk/dataset/the-2016-release-of-the-predicts-database-v1-1. The source data necessary to reproduce all the figures in this study, can be found in the following repository: https://figshare.com/articles/dataset/CarbonFoodNature_TradeOffs_-_Source_data/29618120

Research involving human participants, their data, or biological material

Policy information about studies with human participants or human data. See also policy information about sex, gender (identity/presentation), and sexual orientation and race, ethnicity and racism.

Reporting on sex and gender	The study did not include human participants
Reporting on race, ethnicity, or other socially relevant groupings	The study did not include human participants
Population characteristics	The study did not include human participants
Recruitment	The study did not include human participants
Ethics oversight	The study did not include human participants

Note that full information on the approval of the study protocol must also be provided in the manuscript.

Field-specific reporting

Please select the one below that is the best fit for your research. If you are not sure, read the appropriate sections before making your selection.

☐ Life sciences ☐ Behavioural & social sciences ☒ Ecological, evolutionary & environmental sciences

For a reference copy of the document with all sections, see nature.com/documents/nr-reporting-summary-flat.pdf

Ecological, evolutionary & environmental sciences study design

All studies must disclose on these points even when the disclosure is negative.

Study description	The study was a modelling study that analyzed land use tradeoffs between carbon sequestration, production and biodiversity. The study was done by creating a model using existing data that was downloaded from the sources describe in the data availability statement.
Research sample	It was a modelling study, therefore there was no research sample
Sampling strategy	It was a modelling study, therefore there was no research sample
Data collection	The study was done by creating a model using existing data that was downloaded from the sources describe in the data availability statement.
Timing and spatial scale	The study was done by creating a model using existing data that was downloaded from the sources describe in the data availability statement.
Data exclusions	Appropriate data for the model was chosen and downloaded. No data was excluded and the modelling process is described in the Methods section.
Reproducibility	The data used is described in the data availability statement. The code that was used to create the model and analyse its outputs can be found as described in the code availability statement.

Randomization | This was a modelling study, therefore no randomization was required
Blinding | This was a modelling study, therefore no blinding was required

Did the study involve field work? ☐ Yes ☒ No

Reporting for specific materials, systems and methods

We require information from authors about some types of materials, experimental systems and methods used in many studies. Here, indicate whether each material, system or method listed is relevant to your study. If you are not sure if a list item applies to your research, read the appropriate section before selecting a response.

Materials & experimental systems

n/a	Involved in the study
☒	☐ Antibodies
☒	☐ Eukaryotic cell lines
☒	☐ Palaeontology and archaeology
☒	☐ Animals and other organisms
☒	☐ Clinical data
☒	☐ Dual use research of concern
☒	☐ Plants

Methods

n/a	Involved in the study
☒	☐ ChIP-seq
☒	☐ Flow cytometry
☒	☐ MRI-based neuroimaging

Plants

Seed stocks | No plants were used in this study

Novel plant genotypes | No plants were used in this study

Authentication | No plants were used in this study
